# Restoring the ON Switch in Blind Retinas: Opto-mGluR6, a Next-Generation, Cell-Tailored Optogenetic Tool

**DOI:** 10.1371/journal.pbio.1002143

**Published:** 2015-05-07

**Authors:** Michiel van Wyk, Justyna Pielecka-Fortuna, Siegrid Löwel, Sonja Kleinlogel

**Affiliations:** 1 Institute for Physiology, University of Bern, Bern, Switzerland; 2 Institute for Animal Pathology, Vetsuisse Faculty, University of Bern, Bern, Switzerland; 3 Systems Neuroscience, Bernstein Fokus Neurotechnology and Johann-Friedrich-Blumenbach Institut für Zoologie und Anthropologie, Universität Göttingen, Von-Siebold-Str. 6, Göttingen, Germany; 4 Sensory Collaborative Research Center 889, University of Göttingen, Göttingen, Germany; University of Cambridge, UNITED KINGDOM

## Abstract

Photoreceptor degeneration is one of the most prevalent causes of blindness. Despite photoreceptor loss, the inner retina and central visual pathways remain intact over an extended time period, which has led to creative optogenetic approaches to restore light sensitivity in the surviving inner retina. The major drawbacks of all optogenetic tools recently developed and tested in mouse models are their low light sensitivity and lack of physiological compatibility. Here we introduce a next-generation optogenetic tool, Opto-mGluR6, designed for retinal ON-bipolar cells, which overcomes these limitations. We show that Opto-mGluR6, a chimeric protein consisting of the intracellular domains of the ON-bipolar cell–specific metabotropic glutamate receptor mGluR6 and the light-sensing domains of melanopsin, reliably recovers vision at the retinal, cortical, and behavioral levels under moderate daylight illumination.

## Abstract 2

Expression in retinal ON-bipolar cells of a chimeric protein comprising the light-sensing domains of melanopsin and the intracellular domains of a metabotropic glutamate receptor can restore sight to a mouse model of retinal degenerative blindness.

## Author Summary

Many blind patients have lost the light-sensing photoreceptor cells in their retinas, while deeper retinal cell layers, which normally cannot sense light, remain relatively intact. Promising new “optogenetic” therapies under preclinical evaluation use a modified virus to introduce light-sensitive proteins into surviving retinal cells, turning them into “replacement photoreceptors” and thereby restoring vision. However, several factors limit the feasibility of a clinical optogenetic therapy. For example, current light-sensing proteins only respond to unnaturally high light intensities and employ foreign signaling mechanisms to activate the target retinal cells. To overcome these limitations, we have engineered a cell-tailored light-sensing protein called Opto-mGluR6, which responds to daylight and activates a native signaling pathway within the target cells. Opto-mGluR6 is a chimeric protein composed of the light-sensing domains of the retinal photopigment melanopsin and the ON-bipolar cell–specific metabotropic glutamate receptor mGluR6, which amplifies the signal and ensures retinal responsiveness to daylight. We show that Opto-mGluR6 reliably recovers vision in the mouse, moving towards a clinical optogenetic therapy for patients suffering from photoreceptor degeneration.

## Introduction

About one in 300 people suffer from complete or partial blindness associated with retinal degenerative diseases such as retinitis pigmentosa (RP), age-related macular degeneration (AMD), and diabetic retinopathy. A palette of potential therapies for photoreceptor loss is currently being investigated. One group of technologies targets the very early disease states and aims to slow or stop the photoreceptor degenerative process using either pharmacology [1] or gene replacement therapy [2,3]. However, since vision loss is often only detected at a rather progressed stage of photoreceptor loss, it is difficult to implement such approaches in a clinical context. A second group of potential therapies aims to restore vision after complete photoreceptor loss. These approaches include stem cell therapy [4], electronic prostheses [5], synthetic photoswitchable ligands [6], and optogenetics [7–9]. Fundamental for all of the above approaches is the finding that inner retinal cell layers remain preserved for an extended time period after photoreceptor degeneration, both in human patients and in retinitis pigmentosa mouse models (*rd1*) [10,11]. Out of these therapeutic approaches, optogenetic gene therapy, which selectively introduces genes encoding light-sensitive proteins into surviving retinal cells to act as “replacement light sensors,” holds considerable therapeutic potential: treatment is ambulant, long-lived, and has the theoretical potential to recover high-resolution vision across the entire visual field.

Proof of principle for optogenetic vision recovery using the microbial optogenetic tool Channelrhodopsin-2 (ChR2) was demonstrated in mouse bipolar and ganglion cells [7,9,12,13]. However, ChR2 requires extremely high light intensities to function and extensive molecular engineering has shown that the light sensitivity of ChR2 is limited and can be maximally increased by 1.5 log units [14–16]. In a clinical context, this implies elaborate technical equipment to boost light intensity and a risk of irretrievably damaging the remaining photoreceptors in RP patients. In addition, ChR2 is an alien microbial protein for inner retinal cells without preexisting pathways regulating its activities.

In this study, we introduce a novel optogenetic tool, Opto-mGluR6, engineered to overcome the above-mentioned limitations that hinder the therapeutic application of optogenetics for clinical vision restoration. Opto-mGluR6 is a chimeric all-retinal G-protein-coupled-receptor (GPCR) consisting of the intracellular domains of the ON-bipolar cell—specific metabotropic glutamate receptor, mGluR6, and the light-sensing domains of melanopsin. Melanopsin, which also belongs to the GPCR protein family, is a blue-light-sensitive retinal photopigment (mouse λ = 467 nm) [17] residing in a small subpopulation of intrinsically photosensitive retinal ganglion cells (ipRGCs) that control the pupillary light reflex as well as the circadian rhythm by signaling to the suprachiasmatic nucleus [18]. Similar to insect, cephalopod, and microbial visual pigments, melanopsin was shown to have a bistable nature [19]. In other words, the light-induced isomerization of the retinal chromophore is reversed whilst bound to the opsin. This property makes melanopsin highly resistant to bleaching by strong light and allows successive light activation without a response rundown. Besides being a resident retinal photopigment, melanopsin is activated by moderate daylight as opposed to the laser light intensities that are required for activating the ion channel ChR2. The second component of Opto-mGluR6 is the mGluR6 receptor, which naturally mediates light responses in ON-bipolar cells by coupling the glutamate signal from the photoreceptor cells to TRPM1 cation channels. Also being a GPCR, mGluR6 greatly amplifies the glutamate signal via an intracellular G-protein coupled second messenger cascade [20]. That is, one activated mGluR6 receptor can close many TRPM1 channels. This is in contrast to microbial opsins such as ChR2, which are not able to amplify unitary signals on the protein level. The mGluR6 component enables Opto-mGluR6 coupling to bipolar cell—specific preexisting G-protein complexes, which include regulators of G-protein signaling (RGS) proteins essential for fast signal kinetics [21,22]. By coupling the bistable light switch of the native retinal melanopsin to the intrinsic G-protein complex of mGluR6, Opto-mGluR6 gains an enhanced light response whilst also largely retaining native ON-bipolar cell signaling. We demonstrate that, when expressed in ON-bipolar cells of blind *rd1* mice, Opto-mGluR6 restores retinal and cortical vision within the light intensity range of cone vision.

## Results

### Design of Melanopsin-mGluR6 Chimeras

**Fig 1:**
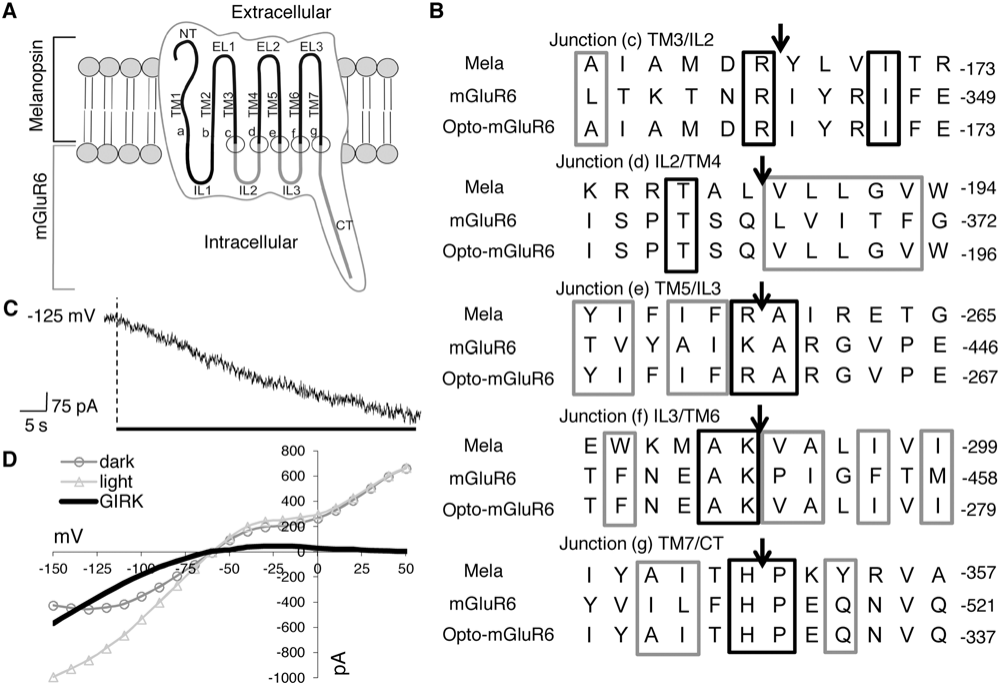
Design of Opto-mGluR6. (A) A sketch of Opto-mGluR6 composed of the N-terminus (NT), transmembrane domains (TM1–TM7), and extracellular loops 1–3 (EL1–EL3) of melanopsin and the intracellular loops (IL) 2 and 3, and the C-terminus (CT) of mGluR6. Locations of the splice sites (connection sites between the melanopsin and mGluR6 peptides) are indicated (a–g). (B) Amino acid sequence alignment of mouse melanopsin, mouse mGluR6, and Opto-mGluR6; splice sites are indicated by arrows (see Supporting Information). Amino acid numbering is indicated to the right. Cages indicate amino acids that are either identical (black) or conserved (grey) on the basis of polarity and acidity. (C) Light response of a HEK293-GIRK cell transiently transfected with Opto-mGluR6 showing the Kir3.1/3.2 induced continuous hyperpolarization in response to 60-s illumination (black bar). (D) Example whole-cell current responses from the same HEK293-GIRK cell to 1-s voltage ramps between -150 mV and +60 mV in the dark and during light stimulation. The subtraction curve (black line) represents the light-induced GIRK current with the characteristic inward rectification of Kir3.1/3.2 channels. See S1 Table for a comprehensive overview of GIRK data.

Melanopsin and mGluR6 both belong to the GPCR family and therefore, despite having little sequence homology, share a highly conserved tertiary structure. To generate Opto-mGluR6, we substituted the second and third intracellular loops (IL2 and IL3) and the C-terminus of melanopsin with that of mGluR6. Previous work has shown that the IL3 of GPCRs is particularly important for G-protein specificity and that this specificity is enhanced by IL2 [23]. The C-terminus was replaced to maintain the protein trafficking and anchoring from wild-type mGluR6 within the ON-bipolar cell. Apart from these modifications, the extracellular and transmembrane (TM) domains, including the chromophore binding pocket of melanopsin [24], were left intact in order to keep Opto-mGluR6 “invisible” to the immune system and to conserve the light-activated photocycle (Fig 1A). Suitable cutting and ligation sites between mGluR6 and melanopsin were primarily based on computer modeling of secondary and tertiary protein structures to identify the borders of intracellular and extracellular domains as well as primary sequence alignment at the N- and C-terminal ends of any particular domain (Fig 1B). We created a total of 11 chimeric melanopsin-mGluR6 variants, all containing the C-terminus of mGluR6 and various IL2 and IL3 replacements with different splice sites (see S1 Text). In all chimeras, the “DRY” motif at the start of IL2, or functional variants thereof (DRIY or NRIY), was conserved. We additionally replaced IL1 in some variants, but this did not improve function (see Supporting Information for details).

### Functionality of Melanopsin-mGluR6 Variants Tested in HEK293 Cells

The activities of the 11 melanopsin-mGluR6 variants were compared using an electrophysiological screen in reconstituted human embryonic kidney (HEK293) cells. The plasmids encoding each of the melanopsin-mGluR6 chimeras (pIRES_melanopsin-mGluR6_TurboFP635) were transiently transfected into a HEK293 cell line stably expressing the G-protein-activated inwardly rectifying K^+^ channel Kir3.1/3.2 (HEK293-GIRK; a kind gift from L. Jan). Group III mGlu-receptors were previously shown to couple to GIRK channels in HEK293-GIRK cells via promiscuous, endogenous G-proteins [25,26]. We illuminated the transfected HEK-GIRK cells with blue light at the peak sensitivity of melanopsin [17], since it has been demonstrated that diverse substitutions of the intracellular loops of the related rhodopsin have no effect on the spectral characteristics of the light switch [23,27,28]. Illumination activated in all 11 melanopsin-mGluR6 variants a GIRK conductance of not significantly different amplitudes, indicating that the splicing sites are not detrimental for function (S1 Table). We selected chimera CM III_ΔL (see Supporting Information for nomenclature) for all subsequent mouse experiments, since it induced the largest mean GIRK conductance (112 ± 40 pS/pF, *n* = 5) and was considered to work effectively. We will refer to this variant as Opto-mGluR6 in the remainder of the manuscript. Fig 1C shows the light-induced hyperpolarization of a HEK293-GIRK cell expressing Opto-mGluR6. Fig 1D depicts the corresponding differential current-voltage relation between basal conditions and illumination, showing an inward rectification with a reversal potential equal to potassium, clearly representing a GIRK current. The differential current at -150 mV was used to calculate the GIRK conductance activated by each chimera (S1 Table). In summary, we showed that melanopsin-mGluR6 chimeric proteins are light activatable and directly couple light stimuli to G-protein signaling.

### Gene Therapy in the Blind *rd1* Mouse Retina

**Fig 2:**
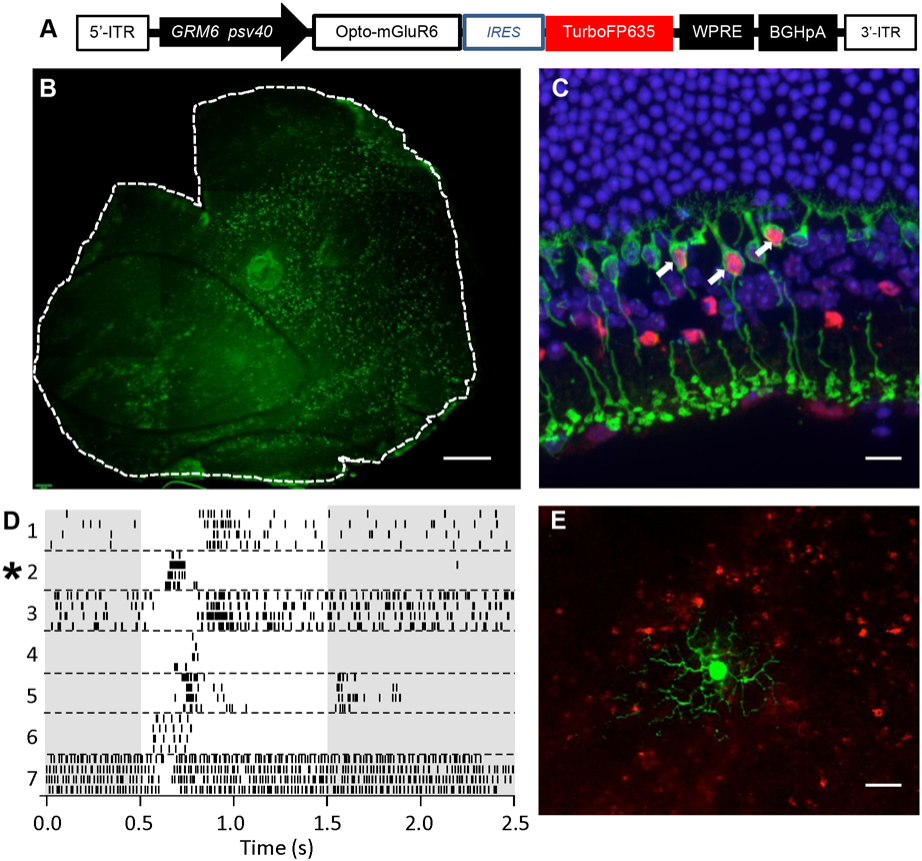
Gene therapy in *rd1* mice. (A) Schematic of the rAAV expression cassette with internal tandem repeats (ITR), the mGluR6 enhancer element (*GRM6*), the sv40 eukaryotic promoter sequence (psv40), an internal ribosomal entry site (IRES), woodchuck posttranscriptional regulatory element (WPRE), and bovine growth hormone polyadenylation sequence (BGHpA). (B) Retinal whole mount transduced by intravitreal injection and stained with an antibody against TurboFP635. The green dots represent transfected ON-bipolar cells. Scale bar, 500 μm. (C) Confocal image of a traverse section through a C57BL/6 retina stained against the rod bipolar cell marker PKCα (green), anti-TurboFP635 (red), and DAPI (blue), showing expressing rod bipolar cells (arrows). Also see S1 Fig. Scale bar, 20 μm. (D) Raster plots of seven RGCs from rAAV_Opto-mGluR6–treated *rd1* retinas showing light responses to 1-s-long blue light pulses (bright band), four traces per cell. Cells are numbered and separated by stippled lines. (E) Fluorescent micrograph of the ganglion cell (green) giving rise to the transient ON response from D (star), with the underlying TurboFP635-labeled bipolar cells shown in red. Scale bar, 40 μm.

To test if Opto-mGluR6 recovers light sensitivity in the retina, we used a recombinant adeno-associated virus (rAAV) to introduce Opto-mGluR6 into the ON-bipolar cells of *rd1* mice. To target expression to ON-bipolar cells but stay within the small packaging limit of rAAV vectors (~4.7 kb), Opto-mGluR6 expression was controlled using the short *GRM6*/sv40 enhancer promoter sequence (Fig 2A) reported to drive gene expression in ON-bipolar cells [9,12,29]. Since wild-type rAAV capsids have a poor ability to transduce bipolar cells, we used a mutated serotype 2 capsid, which was shown to be more effective in transducing inner retinal layers [30]. The packaged rAAVs were injected, subretinally and intravitreally, both into albino FVB/N *rd1* mice (albinos facilitate placement of the injection) and wild-type C57BL/6 mice at ~5–6 wk of age. We observed similar efficiency and distribution of reporter gene (TurboFP635) expression in both subretinally and intravitreally injected retinas 4-wk postinjection (Fig 2B). The majority of strongly expressing cells were ON-bipolar cells, visualized in immunolabeled cryosections of rAAV-infected C57BL/6 retinas (Fig 2C). However, some nonspecific labeling of amacrine and retinal ganglion cells (RGCs) was also found (S1 Fig). Opto-mGluR6 expression lasted up to 8 mo (no longer tested) and did not lead to any observable immunogenic or cytotoxic effects within the retina as shown by immunocytochemistry with antibodies against CD45 (general immune cell marker) and GFAP (astrocyte marker; S2 Fig).

The potential of Opto-mGluR6 to recover light responses in RGCs was assessed 7–34-wk postinjection when nontreated FVB/N littermates were functionally blind (S3 Fig). We targeted RGCs that did not express TurboFP635 and overlay a cluster of expressing bipolar cells. Approximately 22% of RGCs (36 out of 161 cells) responded to light (Fig 2D). The low fraction of RGC responses to light reflects the rather sparse transduction of Opto-mGluR6 into the bipolar cells (12.2 ± 1.5% of overlying ON-bipolar cells expressing in areas of targeted RGCs; Fig 2E). Nonetheless, in line with previous studies expressing ChR2 in retinal ON-bipolar cells [9,12], expression of the light sensor in only a fraction of ON-bipolar cells appeared to suffice to induce light activity in RGCs. Both ON and OFF responses were recorded in RGCs (Fig 2D); however, they had increased response latencies and variability compared to wild-type retinas. Variability between cells is likely a result of the uneven distribution of expressing bipolar cells in the retina (see Fig 2B), whereas variability within RGC responses and the slower response onset are probably because of nonsaturating input (see S4 Fig).

### Functional Evaluation of Opto-mGluR6 in the Mouse Retina

**Fig 3:**
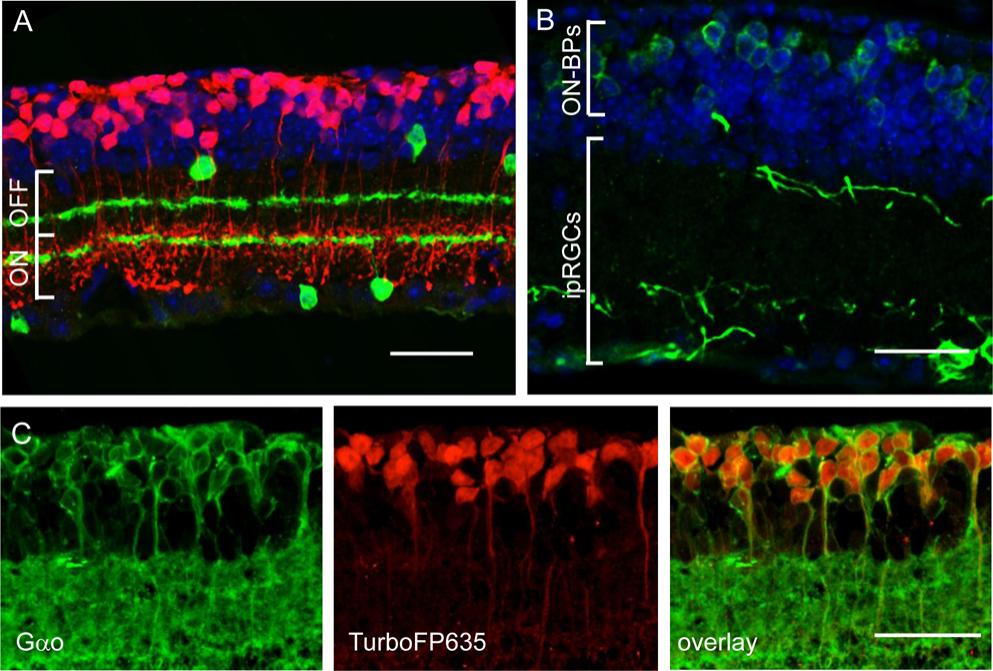
Immunocytochemistry of the *rd1*_Opto-mGluR6 retina. (A) Double labeling against cytoplasmic TurboFP635 (red) and ChAT (green), serving as depth marker in the inner plexiform layer (IPL), subdividing ON and OFF sublaminae (white brackets). All TurboFP635-positive bipolar cells project their axons to the ON-sublamina of the IPL. Nuclei are labeled with DAPI (*blue*). (B) Staining against the N-terminal part of melanopsin labels both ipRGCs and Opto-mGluR6 in the perikarya of ON-bipolar cells (ON-BPs). (C) Double labeling of TurboFP635 (red) with the pan ON-bipolar cell marker Gαo (green) shows a 100% overlay, indicating that all ON-BPs express Opto-mGluR6_IRES_TurboFP635. Scale bars, 20 μm.

Because of the compromised specificity of the *GRM6*/sv40 enhancer promoter and the low viral transduction efficiency, we generated a transgenic *rd1* mouse line that expresses Opto-mGluR6 under the full-length *GRM6* promoter (*rd1*_Opto-mGluR6 line) to evaluate the full potential of Opto-mGluR6 to recover vision. Immunocytochemical staining of melanopsin, TurboFP635, and Gαo in *rd1*_Opto-mGluR6-TurboFP635 retinas confirmed strong expression of both Opto-mGluR6 and the reporter gene TurboFP635 exclusively in all ON-bipolar cells (Fig 3). Opto-mGluR6 expression was restricted to the ON-bipolar cell perikarya (Fig 3B) similar to mGluR6 in age-matched *rd1* retinas [31].

**Fig 4:**
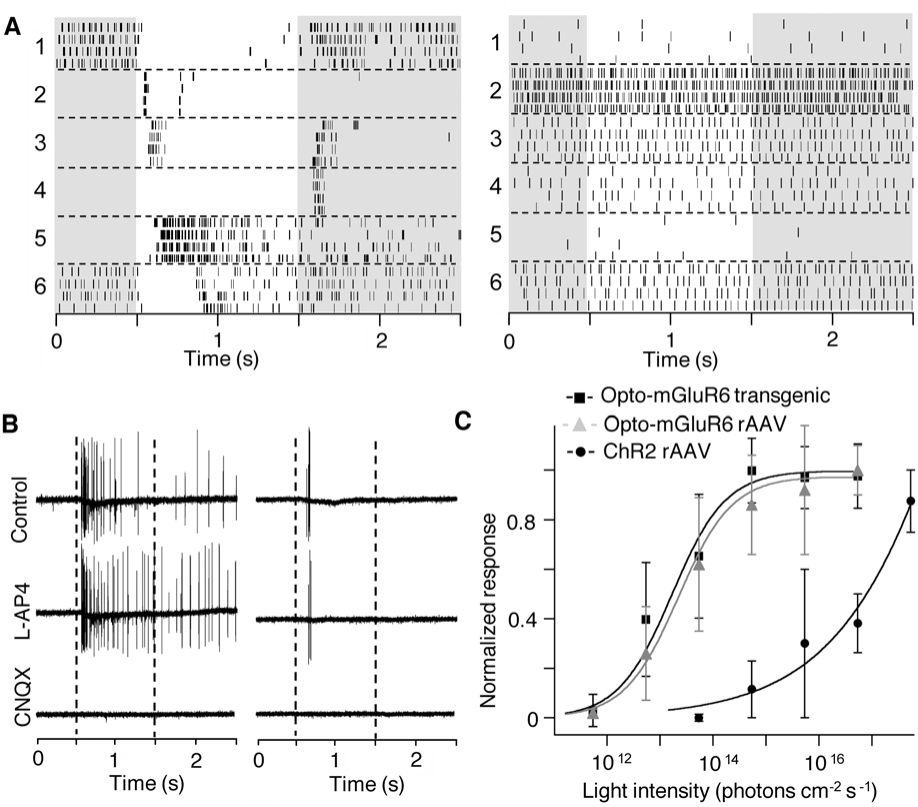
Light responses in *rd1*_Opto-mGluR6 retinas. (A) Despite expression of Opto-mGluR6 only in ON-bipolar cells, the RGCs have both ON and OFF receptive fields (left). RGCs of *rd1* littermates did not respond to the same light stimulus (right). Light steps are indicated by bright underlays. (B) Example light responses of a sustained ON (left) and a transient ON (right) RGC that are not antagonized by 20-μM L-AP4 but are blocked by 10-μM CNQX, indicating that these light-ON responses arise from bipolar cells. Light step is indicated by broken vertical lines. Pharmacology performed on a total of six cells. (C) Average light intensity response curves of seven RGCs from transgenic Opto-mGluR6 retinas and six RGCs from rAAV-transduced Opto-mGluR6 retinas compared to published values for ChR2 [9,12]. The Opto-mGluR6 response saturated at light intensities required for ChR2 activation. The light-induced changes in spike rate were normalized to a maximum of 1 for each RGC (see S4 Fig for details).

We assessed the blue-light-driven activity of RGCs in p170–p320 old transgenic *rd1_*Opto-mGluR6 mice and their nontransgenic age-matched littermates. We observed robust light responses in *rd1-*Opto-mGluR6 mice, which were completely absent in nontransgenic littermates (Fig 4A). In contrast to wild-type retinas, responses in *rd1-*Opto-mGluR6 retinas were insensitive to 20 μM L-(+)-2-amino-4-phosphonobutyric acid (L-AP4; Fig 4B), an agonist of the native mGluR6 receptor, confirming that light responses did not originate from residual photoreceptors. The same responses were blocked by 10 μM of the AMPA/kainate receptor antagonist 6-cyano-7-nitroquinoxaline-2,3-dione (CNQX; Fig 4B), showing that the light responses also did not arise from melanopsin-expressing ipRGCs.

The visual system covers a very broad light intensity range from 10^4^ to 10^16^ photons cm^-2^ s^-1^ [32]. Optogenetic vision recovery in mice expressing ChR2 in ON-bipolar cells reported a light intensity threshold for RGC spiking as high as 10^15^–10^16^ photons cm^-2^ s^-1^ [9,12]. Such high light intensities are only encountered in the brightest natural conditions, such as a bright sunny day on a snowfield [33], and are over extended periods phototoxic to the retina [34]. To examine the relative light sensitivity of Opto-mGluR6, we generated light-intensity-response curves for RGCs in the *rd1*_Opto-mGluR6 transgenic retina as well as the rAAV-transduced *rd1* retina (see S4 Fig for a description of the method) and compared them to the RGC intensity-response values reported previously for ChR2. The minimum light intensity required (i.e., threshold) to evoke RGC responses in Opto-mGluR6-expressing retinas was ~5 x 10^11^ photons cm^-2^ s^-1^ compared to ~10^15^ photons cm^-2^ s^-1^ previously reported for ChR2 (Fig 4C) [9,12]. Opto-mGluR6 light responses saturated at ~5 x 10^14^ photons s^-1^ cm^-2^, which is the activation threshold for ChR2 (Fig 4C). Specifically, the light intensities inducing half-maximal RGC responses were 1.6 x 10^13^ photons s^-1^ cm^-2^ for the *rd1*_Opto-mGluR6 transgenic retina, 2.3 x 10^13^ photons s^-1^ cm^-2^ for the rAAV-transduced *rd1* retina, and 5 x 10^16^ photons s^-1^ cm^-2^ for the ChR2-expressing retina. Importantly, the light-response curves for the transgenic and rAAV-transduced Opto-mGluR6 retinas were almost identical, proving that Opto-mGluR6-mediated responses are truly more light-sensitive than those mediated by ChR2. In other words, it is not the panretinal Opto-mGluR6 expression in the transgenic mouse retina that increased the light-sensitivity of Opto-mGluR6 compared to the reported ChR2 sensitivity determined in low-expressing rAAV-transduced retinas; instead, the increased light sensitivity of Opto-mGluR6 arises from intracellular signal amplification mediated through the G-protein. In line with the lower Opto-mGluR6 expression in rAAV-treated retinas, RGCs from rAAV-retinas reached only one-third of the average maximum spike count determined in transgenic retinas.

The dynamic range of RGC activity (increase of firing rate until saturation) mediated by Opto-mGluR6 covered ~2.5 log units of light intensities and is equivalent to the reported dynamic range of RGC responses in the wild-type retina [35], indicating that inner retinal processing is maintained. In conclusion, Opto-mGluR6 expression in ON-bipolar cells drives robust light-evoked RGC responses in the natural luminance range of cone vision, thereby overcoming one of the major shortfalls of traditional optogenetic tools for vision restoration.

### Characterization of Opto-mGluR6 in the mouse retina

**Fig 5:**
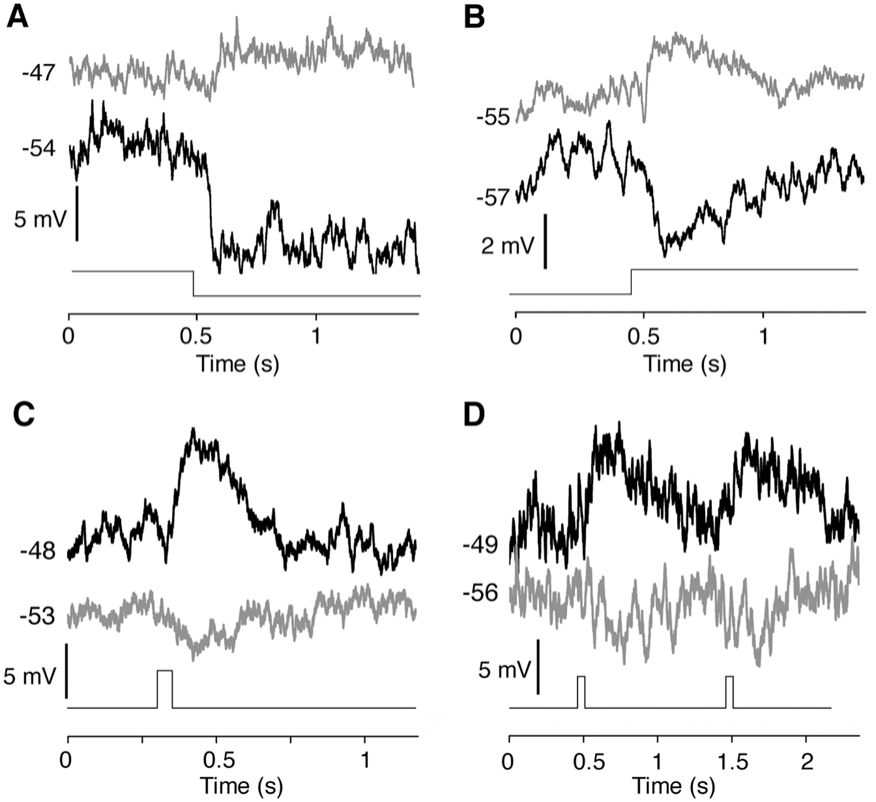
Example traces of direct patch-clamp recordings from bipolar cells demonstrating inverse response polarity of Opto-mGluR6 compared to photoreceptor-activation without changes in response kinetics. We recorded from 21 bipolar cells in dark-adapted *Pde6b* ^+^_Opto-mGluR6 retinal slices before (black traces) and after (grey traces) the photoreceptor input was eliminated by bleaching and application of L-AP4. (A,B) Bipolar cell responses to a long (2 s) light pulse. The native ON-bipolar cell hyperpolarized to a light-OFF stimulus (step down at 0.5 s) but depolarized to the same stimulus under direct Opto-mGluR6 activation (A). Conversely, the native OFF-bipolar cell responded with hyperpolarization to a light-ON stimulus (step up at 0.5 s) but depolarized to the same stimulus under Opto-mGluR6 activation (B). (D,E) ON-bipolar cell responses to short (50 ms) light stimuli. Opto-mGluR6-mediated responses have an identical time course to the photoreceptor-mediated response under inversion of response polarity. Opto-mGluR6 reliably encodes multiple short light pulses (D).

**Fig 6:**
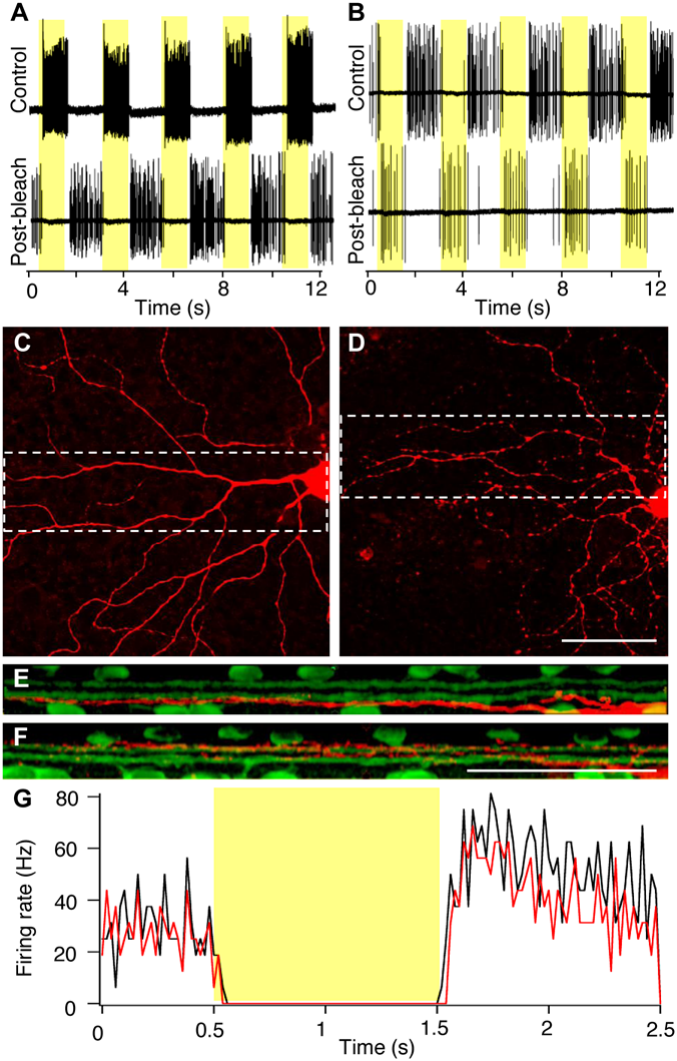
Opto-mGluR6 flips the polarity of light responses in RGCs. (A) Example spike trains recorded from an ON-Alpha RGC in an isolated *Pde6b* ^+^_Opto-mGluR6 retina in response to a series of light steps (yellow underlay, 5.4 x 10^16^ photons cm^-2^ s^-1^, 100% contrast, yellow highlights), before (top trace) and after (bottom trace) the photoreceptor cells were bleached. (B) The same experiment from panel A performed on an OFF-Alpha cell. After recording, cells were labeled with neurobiotin (red). Panels (C,D) show flat views of the cells recorded from in A and B, respectively. (E,F) show transverse views of the marked areas in C and D, respectively. Colabeling with ChAT (green) confirms the identity of the ON- and OFF-Alpha cells on the basis of their dendritic stratification depth. Scale bars, 50 μm. (G) Spike-time histograms comparing the response kinetics of OFF-Alpha cells from C57BL/6 retinas (black) with that of ON-Alpha cells from bleached *Pde6b* ^+^_Opto-mGluR6 retinas (red). Each trace shows the average of eight traces recorded from four cells from four retinas, bin width 20 ms.

RGCs of *rd1-*Opto-mGluR6 retinas had both ON and OFF (and ON-OFF) receptive fields (Fig 4A). This reflects the well-characterized crosstalk from ON- to OFF-bipolar cells via the AII amacrine cell network, explained in S5 Fig. AII amacrine cells receive direct glutamatergic input from rod bipolar cells [36] and indirect input from cone ON-bipolar cells via gap junctions [37–39]. This ON signal is then relayed to the OFF-bipolar cells via a sign-inverting glycinergic synapse. Since native mGluR6 is activated in the dark (photoreceptors release glutamate in the dark), but Opto-mGluR6 is directly activated by light, all Opto-mGluR6-expressing native ON-bipolar cells in the treated *rd1* retina are expected to turn into OFF cells. If the AII amacrine circuitry is functional, Opto-mGluR6-expressing bipolar cells provide negative feedback to the terminals of the native OFF-bipolar cells and create a second group of bipolar cells with an ON response (native OFF cells). Because of the sign inversion, one would expect an inversion of response polarity in bipolar cells when light activation is mediated through Opto-mGluR6 as opposed to photoreceptors. To test this, we performed direct patch-clamp recordings from bipolar cells (*n* = 21) in retinal slices of seeing *Pde6b* ^+^_Opto-mGluR6 mice (see Methods for mouse nomenclature). Seeing *Pde6b* ^+^_Opto-mGluR6 mice were chosen to enable a side-by-side comparison of photoreceptor and Opto-mGluR6–mediated responses in the same bipolar cell. After the establishment of a stable recording, the bipolar cell was activated with a reduced-intensity 465-nm light pulse (1 x 10^11^ photons cm^-2^ s^-1^) in order to avoid coactivation of Opto-mGluR6. The photoreceptor input to the patched bipolar cell was subsequently eliminated by photopigment bleaching with high-intensity white light (5 min, 5.35 x 10^17^ photons cm^-2^ s^-1^) [40] and additional pharmacological block of the photoreceptor-to-bipolar cell synapse with L-AP4. Then the light response of the same bipolar cell was again recorded, but this time to an increased light intensity in order to fully activate Opto-mGluR6 (5 x 10^16^ photons cm^-2^ s^-1^), which is not affected by bleaching. Fig 5 demonstrates the inverse response polarity of Opto-mGluR6 compared to photoreceptor activation. The ON-bipolar cell depicted in Fig 5A hyperpolarized to a light-OFF stimulus but depolarized to the same stimulus after elimination of the photoreceptor input, now being directly activated by Opto-mGluR6. Conversely, the OFF-bipolar cell depicted in Fig 5B responded with hyperpolarization to a light-ON stimulus but depolarized to the same stimulus under Opto-mGluR6 activation. These results confirm the integrity of the AII amacrine network described above (S5 Fig). The findings were corroborated by recordings from ON-Alpha (*n* = 5) and OFF-Alpha (*n* = 5) RGCs in the same setting. ON-Alpha RGCs were chosen because they are known to receive direct input exclusively from ON-bipolar cells [39], which allowed us to consistently target the same bipolar cell population. Furthermore, they are easy to target for recordings because of their large cell bodies and characteristic light responses. Fig 6A shows that a light-ON stimulus induces ON-Alpha cell firing in the isolated dark-adapted retina. After elimination of the photoreceptor response, the polarity of the light response inverted (now responding at light OFF, characteristic of an OFF-Alpha cell). Equally, light responses of OFF-Alpha cells inverted to respond at light ON under Opto-mGluR6 activation (Fig 6B). This inversion of response polarity at the RGC level was further confirmed by morphological analysis subsequent to recordings. In line with the above results, RGCs that showed an OFF response under Opto-mGluR6 activation had a clear ON morphology and vice versa (Fig 6C–6F). Our results not only demonstrate response polarity inversion at the bipolar and RGC levels but also the conservation of a functional inner retinal circuitry mediating both ON and OFF responses. Furthermore, the resistance of Opto-mGluR6 to bleaching by extensive illumination verifies the bistable nature of melanopsin.

The above experiments also allowed us to directly resolve and compare the kinetic latencies mediated by photoreceptors and those mediated by Opto-mGluR6. The Opto-mGluR6 kinetics of the RGC responses (latency to first spike of 73 ± 33 ms) nearly perfectly matched those mediated through photoreceptors (84 ± 18 ms; *p* = 0.229, *t* test; Fig 6G). The direct Opto-mGluR6 latencies were apparent from bipolar cell recordings to short 50-ms light stimuli. Although bipolar cell recordings are noisy and response kinetics not fast enough to follow short light stimuli precisely [41], Fig 5 clearly shows that kinetic latencies do not differ between photoreceptor- and Opto-mGluR6-mediated responses. ON latencies to maximal response amplitude were typically 25–50 ms in bipolar cells, whereas OFF kinetics were slightly slower, with an average latency of approximately 100 ms. Fig 5D shows that repetitive stimuli are equally well encoded by Opto-mGluR6 and photoreceptors. These results confirm that the melanopsin light switch of Opto-mGluR6 is intrinsically fast and that the sluggish light responses observed in ipRGCs and other cells heterologously expressing melanopsin (HEK293-GIRK cells expressing Opto-mGluR6) [42] are an effect of the dynamics of the coupled G-protein and intracellular signaling cascade. Thus, the kinetics of Opto-mGluR6-mediated activation and deactivation of bipolar cells closely resembles that of native mGluR6, which is considered a prerequisite for the recovery of normal vision.

**Fig 7:**
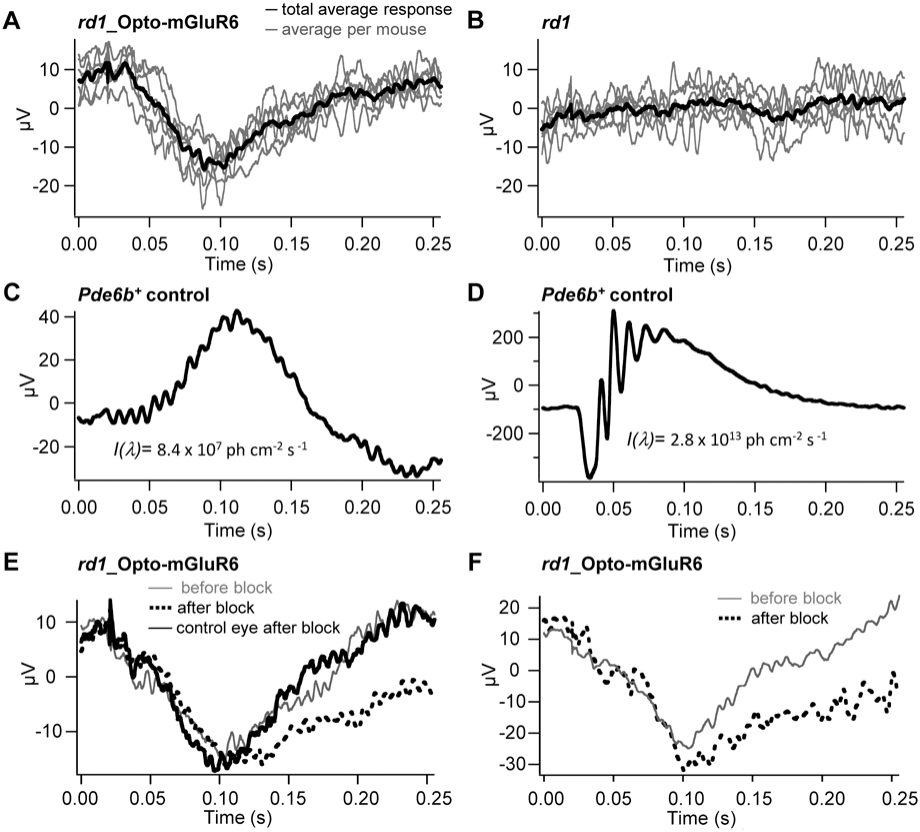
ERGs of *rd1*_Opto-mGluR6 mice show an inverse, electronegative b-wave. (A) Negative deflection in response to a strobe flash measured in *rd1*_Opto-mGluR mice. (B) Blind *rd1* littermates had no detectable ERG. (C,D) The ERGs of seeing *Pde6b* ^+^ mice to a low-intensity (C) and high-intensity (D) strobe flash. ERGs of nonsaturating light intensities (C) lack oscillatory potentials and show slowed b-wave kinetics compared to ERGs of saturating light intensities (D). (E,F) Pharmacology on the electronegative ERG depicted in (A), before (grey traces) and after (broken traces) intravitreal injection of inhibitors. Application of DL-APV and L-AP4 slowed the response kinetics (E), and additional application of CNQX increased the ERG amplitude (F). The black trace in (E) shows the ERG of the control (left) eye after the inhibitor mix has been injected into the contralateral (right) eye; no change in the ERG is visible in this internal control.

To further characterize Opto-mGluR6-mediated signaling in *rd1*_Opto-mGluR6 mice, we performed baseline electroretinogram (ERG) measurements. The scotopic ERG recordings from *rd1_*Opto-mGluR6 mice (p270–p290) clearly showed an ERG response to a white strobe flash (2.8 x 10^13^ photons cm^-2^ s^-1^) that was absent in blind *rd1* littermates (Fig 7A and 7B). The shape and amplitude of the Opto-mGluR6-mediated ERG looked similar to the attenuated scotopic b-wave of dark-adapted, seeing *Pde6b* ^+^ control mice at light intensities that were ~5 log units lower (8.4 x 10^7^ photons cm^-2^ s^-1^), albeit with an inverse polarity (Fig 7C and 7D). The response attenuation is explained by the used strobe intensity, which corresponds to the Opto-mGluR6 half-saturation intensity (compare Fig 4C), whilst the scotopic rod half-saturation intensity lies at values that are ~5 log units lower. Since it is generally accepted that ON-bipolar cell activity is the main generator of the b-wave [43,44], it is not surprising that the observed ERG follows the response kinetics of the scotopic b-wave. The inversion of the ERG is interesting and may be predicted by the bipolar cell response polarity inversion described above. In order to characterize the observed ERG in more detail, we used pharmacology. Blocking the photoreceptor to ON-bipolar cell synapse with intravitreally injected L-AP4 did not change the ERG, as expected from the lack of an ERG in nontransgenic littermates (Fig 7B). Therefore, the observed electronegative ERG must be generated from Opto-mGluR6-induced activity in ON-bipolar and thereto postsynaptic cells. To tease out potential contributions from amacrine and RGCs, we applied the *N*-Methyl-D-aspartic acid (NMDA)-receptor antagonist DL-2-Amino-5-phosphonopentanoic acid (DL-APV), which has been shown to abolish contributions from third-order neurons [45]. The ERG persisted, albeit also with decelerated recovery to baseline (Fig 7E), confirming that bipolar cells are the generators of the b-wave but that negative feedback loops from third order neurons naturally truncate the observed ERG [46]. Since it was proposed that the ON-bipolar cell—generated b-wave is opposed by OFF-bipolar cell activity [44], we additionally blocked the photoreceptor to OFF-bipolar cell synapse with CNQX. In line with the proposed push-pull model of ON- and OFF-bipolar cells, we observed a small increase in the measured b-wave (Fig 7F). The experiments depicted in Fig 7E and 7F not only prove that the observed electronegative ERG is generated by Opto-mGluR6-mediated ON-bipolar cell activity but also confirm that the inner retinal AII amacrine circuitry is functional. They also once again illustrate the sign inversion of bipolar cell signaling under Opto-mGluR6 activation.

### Recovery of Vision beyond the Retina

To assess if Opto-mGluR6 can recover vision beyond the retina in the *rd1* mouse, we performed *in vivo* optical imaging of the primary visual cortex (V1) and conducted two behavioral tests. We used an optokinetic reflex drum [47] to test for integrity of the subcortical reflex circuitry and a cued visual water task to assess perceptual vision.

**Fig 8:**
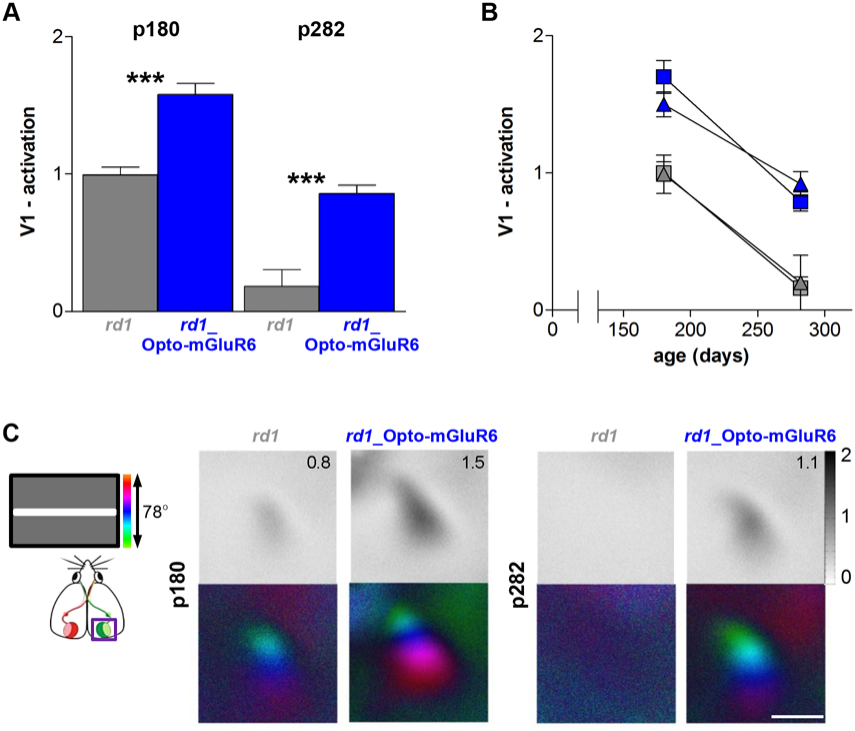
*rd1*_Opto-mGluR6 mice show significantly higher V1 activation compared to *rd1* littermates both at p180 and p282. (A,B) Quantification of V1 activation of the right and left primary visual cortex (V1) from *rd1* (grey, p180: *n* = 5; p282: *n* = 4) and *rd1*_Opto-mGluR6 mice (blue, p180 *n* = 3; p282, *n* = 4), plotted separately for the two ages (A) or as a function of age (B) for V1 activation of the left (squares) and right (triangles) hemispheres (mean ± SEM, ANOVA, ***p < 0.001). (C) Typical examples of binocular V1 activity maps of *rd1*_Opto-mGluR6 mice and *rd1* littermates. The grey-scale coded response magnitude maps (upper rows) are illustrated as fractional change in reflection x 10^-4^. Retinotopic maps (lower rows) are color-coded according to the scheme on the left. Scale bar, 1 mm.

The magnitude of visually driven activity in V1 of *rd1_*Opto-mGluR6 mice and their nontransgenic *rd1* littermates was evaluated at two different adult ages, ~6 mo (on average p180) and ~9 mo (on average p282), by in vivo optical imaging of blood oxygen [48]. We recorded V1 activation during binocular visual stimulation from both hemispheres of individual mice (Fig 8). In both age groups, V1 activation of all *rd1*_Opto-mGluR6 mice was significantly higher than that of *rd1* mice, with nonoverlapping distributions (Fig 8A and 8B). We still recorded reliable stimulus-driven V1 activation in all *rd1* mice at 6 mo of age with responses approaching zero at 9 mo of age (Fig 8A–8C). Quantification of the imaging data (Fig 8A and 8B) revealed that at 6 mo of age, V1 activation of the *rd1*_Opto-mGluR6 mice was 1.7 ± 0.1 (mean ± standard error of the mean [SEM], *n* = 3) in the left and 1.5 ± 0.1 in the right V1 (*n* = 3) compared to 0.9 ± 0 (*n* = 5) in the left and 0.9 ± 0.1 (*n* = 4) in the right V1 in *rd1* mice (ANOVA, *p* < 0.005 for left V1; *p* < 0.01 for right V1). At 9 mo of age, V1 activation of the *rd1*_Opto-mGluR6 mice was 0.8 ± 0.1 in the left V1 (*n* = 4) and 0.9 ± 0.1 in the right V1 (*n* = 4), significantly higher than in *rd1* mice in which V1 activation approached zero (0.2 ± 0.2 in the left V1 and 0.2 ± 0.2 in the right V1, both *n* = 4, ANOVA, *p* < 0.005 for left V1; *p* < 0.01 for right V1).

**Fig 9:**
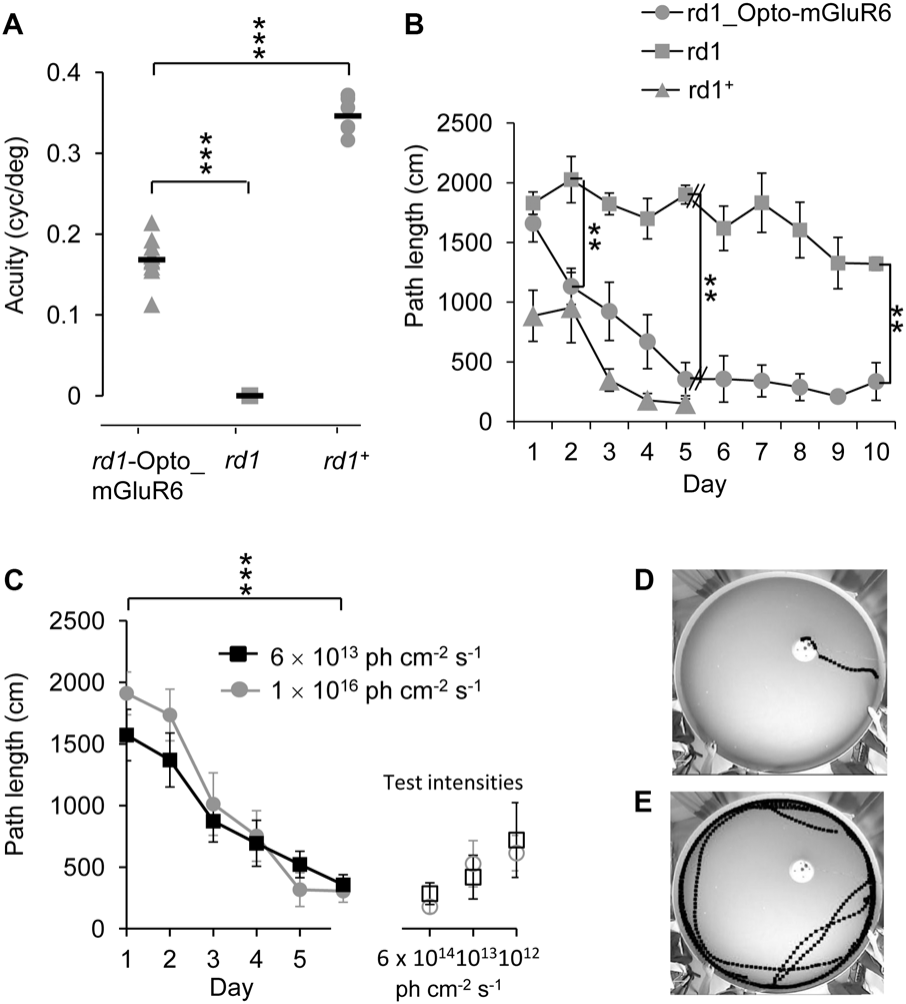
Visual performance determined in behavioral experiments. (A) *rd1*_Opto_mGluR6 mice (*n* = 11) had optokinetic reflexes with a peak spatial acuity of 0.17 ± 0.04 cyc/deg, *rd1* littermates (*n* = 8) did not respond to any frequency, and seeing *Pde6b* ^+^ mice (*n* = 6) responded with a peak acuity of 0.35 ± 0.05 cyc/deg. Stimulus intensity 5.6 × 10^13^ photons cm^-2^ s^-1^. (B) The total swim distance to a hidden platform in a water maze marked by an overhead blue light-emitting diode (LED). From training day 2, *rd1*_Opto-mGluR6 mice (*n* = 6) learned to find the platform significantly faster than their blind *rd1* littermates (*n* = 5) but not significantly slower than *Pde6b* ^+^ mice (*n* = 3). Stimulus intensity 1 × 10^16^ photons cm^-2^ s^-1^. (C) Sensitivity of behavioral vision. *rd1*_Opto-mGluR6 mice were trained for 6 d at high and low light intensities (left) and subsequently tested for their visual performance at three test intensities (right). Nonsaturating training light intensities did not compromise learning, and the performance at the three test intensities did not differ significantly. Error bars indicate SEM in B and C. (D,E) Example paths swam by *rd1*_Opto-mGluR6 and *rd1* mice, respectively, on training day 10.

We next compared the optomotor performance of transgenic *rd1*_Opto-mGluR6 mice (p175–p196), nontransgenic *rd1* littermates and age-matched seeing *Pde6b* ^+^ control mice (Fig 9A). *rd1*_Opto-mGluR6 mice showed clear visual tracking behavior with a peak spatial frequency of 0.17 ± 0.04 cycles per degree (cpd) and performed significantly better than blind *rd1* littermates that displayed no optomotor reflex as was shown previously [49]. The Opto-mGluR6-mediated visual acuity was improved compared to values measured in microbial-opsin-treated *rd1* mice [8]; however, values still remained significantly below the values determined for seeing *Pde6b* ^+^ mice (0.35 ± 0.06 cpd; *p* < 0.001, *t* test). Part of this discrepancy may be due to the relatively low stimulating light intensity of the visual drum (~5 x 10^13^ photons s^-1^ cm^-2^), which only half saturates Opto-mGluR6 responses (see Fig 4C) whilst saturating rod responses.

Although optokinetic responses provide information about responsiveness to spatially organized stimuli, the optokinetic reflex does not require cortical circuits and the quality of perceptual vision remains unclear. Thus, we designed a visually guided swim task in which mice were required to find a submerged escape platform in the pool signaled by an overhead LED with Opto-mGluR6 saturating intensity (1 x 10^16^ photons s^-1^ cm^-2^). The length of the swim paths required to find the hidden platform were measured over 10 consecutive days of training in six *rd1*_*Opto-mGluR6* mice (p270) and five age-matched *rd1* littermates. Three age-matched *Pde6b* ^+^ mice served as positive controls and were trained for only 5 consecutive days, after which they reliably found the platform. After 5 days of training, *rd1*_Opto-mGluR6 mice were able to directly swim to the platform (path length 355 ± 139 cm, mean ± SEM) and performed not significantly worse compared to control *Pde6b* ^+^ mice (151 ± 40 cm, mean ± SEM, Fig 9B and 9D). The *rd1* mice, on the other hand, performed significantly worse than *rd1*_Opto-mGluR6 mice; after 10 days of training, their swimming paths were ~4-fold longer than those of *rd1*_Opto-mGluR6 mice and not platform aimed (1321 ± 56 cm, mean ± SEM, *p* = 0.002, Fig 9B and 9E). It should be noted that *rd1* mice still slightly shortened their path length over the training period (Fig 9B), probably because mice learned to swim away from the tank´s edge and/or because ipRGCs support visual navigation [50,51]. To investigate if subthreshold light intensities would compromise behavioral vision, we trained three *rd1*_Opto-mGluR6 mice at reduced light intensities, similar to the intensity used in the optokinetic reflex (OKR) (~5 x 10^13^ photons s^-1^ cm^-2^), and compared their performance to three *rd1*_Opto-mGluR6 mice that were trained at high light intensities used initially (1 x 10^16^ photons s^-1^ cm^-2^). Remarkably, attenuation of the light intensity did not compromise learning in the visually guided swim task (Fig 9C). Therefore, the reduced visual acuity of *rd1*_Opto-mGluR6 mice in the OKR experiments cannot confidently be attributed to insufficient Opto-mGluR6 activation. To figure out the sensitivity of behavioral vision, we subjected the mice trained at “high” and “low” light intensities to test trials at attenuated light intensities, including the threshold activation intensity of Opto-mGluR6 determined in RGCs before (6 x 10^12^ photons s^-1^ cm^-2^, see Fig 4C). The performance of the mice again did not significantly differ at any of the test intensities (Fig 9C), suggesting that cortical vision is remarkably sensitive to subsaturated retinal output.

## Discussion

An ideal therapy for patients suffering from photoreceptor degeneration will not only restore the light sensitivity of the retina but (i) will also function at environmental light intensities, (ii) will be physiologically compatible with the surviving inner retina, (iii) will conserve a natural range of RGC trigger features, and (iv) will be devoid of toxic and immunogenic side effects. All of this should be accomplished with a minimally invasive and safe clinical technology. Opto-mGluR6, which overcomes most shortfalls of existing optogenetic tools, meets most of these criteria and enhances the clinical feasibility of optogenetic vision recovery.

We showed that Opto-mGluR6 targeted to retinal ON-bipolar cells of mice suffering from photoreceptor degeneration not only recovers light sensitivity in RGCs at moderate light intensities but also reestablishes diverse RGC light responses comprising ON, OFF, ON-OFF, sustained, and transient responses. This suggests that Opto-mGluR6 is able to sufficiently drive ON-bipolar cells and thereto postsynaptic third-order neurons to activate the inhibitory amacrine feedback loops dissecting the ON-bipolar cell signal into ON, OFF, transient, and sustained components [46].

Opto-mGluR6 is a light-activated metabotropic receptor and able to amplify light signals through the preexisting G-protein-coupled enzymatic cascade of mGluR6. That Opto-mGluR6’s increased light sensitivity indeed arises from intracellular signal amplification and not simple convergence of more expressing bipolar cells in the transgenic mouse as opposed to experiments with rAAV-transduced mice is apparent from Fig 4C. The ion channel ChR2 on the other hand has a very small unitary conductance (~60 fS) [52], and high expression levels are needed to sufficiently depolarize bipolar cells and activate inner retinal circuitries. As a result, the recovery of indirect OFF-RGC responses (in addition to ON responses) required very high ChR2 expression levels [53,54]. Opto-mGluR6 is over three orders of magnitude more light sensitive than ChR2 and recovered even in our low-expressing rAAV system both ON and OFF responses. The idea of light signal amplification through G-protein coupling was pioneered by Lin et al. (2008), who used unmodified melanopsin ectopically expressed in a large number of RGCs (and other inner retinal cells) in *rd1* mice. Whilst they achieved similar RGC light sensitivity to Opto-mGluR6, which complies with the sensitivity of ipRGCs [55,56], the RGC responses recorded by Lin and colleagues were all of the ON type, variable and slow (in the order of seconds) [13]. In contrast, responses in Opto-mGluR6-expressing retinas were of both ON and OFF type, with fast kinetics similar to light responses recorded from healthy wild-type retinas. This suggests that Opto-mGluR6 assembles with the preexisting G protein/RGS/TRPM1-complexes of native mGluR6 to mediate fast response kinetics [21,22]. Another interesting recent approach to generate light-activatable mGluRs used synthetic photoswitchable ligands [26]. Although this approach shares some of the advantages of Opto-mGluR6, synthetic photoswitches depend on continuous external supplementation of the synthetic ligand, limiting their usefulness in a clinical context.

By targeting the tightly spaced cell bodies of bipolar cells, we not only took advantage of signal amplification of the metabotropic receptor cascade within the bipolar cells and the neuronal convergence from bipolar to RGCs but also achieved a relatively high photon catch [57]. The minimal light intensity required to stimulate RGCs in an Opto-mGluR6-expressing retina (~10^11^ photons s^-1^ cm^-2^) is in the range of the threshold intensity for cone vision [32]. In contrast, ChR2 only starts responding at light intensities equivalent to a sunny day on a snowfield [33]. Although novel ChR2 variants with increased light sensitivity exist [14,16], they are still markedly (1–2 log units) less light sensitive than Opto-mGluR6 and carry all the limitations of microbial proteins. The relatively high light sensitivity of Opto-mGluR6-mediated responses is a clear advantage over ChR2 regarding a potential clinical application. Opto-mGluR6 can be activated by daylight and does not require elaborate technical equipment such as intensifier goggles and image converters. Although phototoxicity is known to mainly affect photoreceptors [58], it should be considered in a clinical context. RP patients often possess remnant cones in the fovea [8] that may be preserved by pharmacological and microRNA (miRNA) treatment [3] conducted concurrently with an Opto-mGluR6 therapy of the inner retina. Another advantage of Opto-mGluR6 is its high physiological compatibility. The extracellular domains originate from melanopsin, which is a native retinal protein and thus nonimmunogenic. The intracellular parts originate from mGluR6, which is the ON-bipolar specific glutamate receptor. Therefore, protein turnover, transport, and activity within the ON-bipolar cell are controlled through preexisting mechanisms, decreasing the risk of a disruption of the healthy cell metabolism [59]. Although we have not observed acute cytotoxic or immunogenic responses in rAAV-Opto-mGluR6-treated mice and these reactions are not foreseen, long-term safety has to be carefully evaluated before Opto-mGluR6 can be considered for clinical use.

The cortical imaging data clearly show that visually driven V1 activation is reliably present in both 6- and 9-mo-old *rd1*_Opto-mGluR6 mice, while only 6-mo-old *rd1* mice had weak but still detectable V1 activation. Although cortical imaging data are in line with the behavioral vision tests in the fact that transgenic mice performed significantly better than nontransgenics, the longevity of V1 light responsiveness in *rd1* mice was somehow unexpected since we measured neither RGC nor OKR responses in *rd1* mice aged ~6 mo (Fig 8A, S3 Fig). However, the present imaging data are perfectly in line with a previous report showing cortical responses in *rd1* mice up to an age of p240 [60]. Similar to previous studies [8,60], we found remnant cones in the retinas of 6- and 9-mo-old *rd1* mice, reduced in number in the older mice and all with atypical morphologies lacking outer segments (S6A and S6B Fig). Since such atypical cones have been shown to no longer confer light sensitivity to the retina [8], we do not consider them to be the source of the remnant V1 activation. We instead propose that the residual activity originates in ipRGCs, which have been shown to drive V1 activity in *rd/rd cl* mice that lack rods and cones entirely [51]. Furthermore, it was shown that mice lacking melanopsin have behavioral deficits in contrast sensitivity and that melanopsin responses are driven at lower light intensities than previously appreciated [61]. All of this suggests that ipRGCs would be activated in our optical imaging experiments and could be the source of the remnant retinotopic V1 activity. However, immunolabeling indicated no difference in the number of ipRGCs in the retinas of 6- and 9-mo-old *rd1* mice (S6 Fig), and it is therefore unclear why the V1 response disappears at p282. One possibility is that input from ipRGCs alone is too weak to maintain the structure of the cortical maps, which are known to have some plasticity in adult mice [62].

Despite the advantages of Opto-mGluR6, vision recovery could still be further improved. Similar to other optogenetic tools, Opto-mGluR6 is not able to restore color vision or the full light-adaptive range of the retina, which is largely mediated by the retinal horizontal cells that are bypassed when activating bipolar cells directly. Bypassing horizontal cells may, however, also be advantageous since neural remodeling in the degenerating retina has been reported to affect—besides photoreceptors—mainly horizontal cells [11]. Since we observed that the overall RGC responses in *rd1*-Opto-mGluR6 mice were often weaker and of a more transient nature than in seeing mice, whilst they were similar in *Pde6b* ^+^_Opto-mGluR6 mice, additional physiological and anatomical changes seem to affect the degenerating inner *rd1* retina. In favor of a bipolar cell rescue approach are reports that bipolar cell axon terminals in the degenerating retina maintain their structure [9] and that activation of ON-bipolar cells fosters the colocalization of mGluR6 signal cascade elements necessary for TRPM1 activity [63]. However, it has been reported that bipolar cells in the *rd1* retina rest at relatively hyperpolarized potentials, which indicates that the mGluR6 receptor cascade is either down-regulated or desensitized [64]. This is consistent with chemical labeling studies that suggested permanent closure of the TRPM1 channel in *rd1* mice [65]. It has also been suggested that cone ON-bipolar and AII amacrine cells produce self-generated oscillations in the degenerating retina that could potentially deteriorate the visual signal [64]. Both of the above could potentially decrease RGC responses in *rd1*-Opto-mGluR6 mice. However, as still little is known about the changes occurring in inner retinal neurons after photoreceptor loss, it is mandatory to consider results from *rd1* mice and potentially other *rd* lines with caution, particularly in regard to drawing conclusions for a therapy in human patients. In this respect, Opto-mGluR6 may serve as a useful tool to investigate inner retinal reorganization during progressing degeneration in *rd* mouse models in more detail, providing us with more robust knowledge. Another intriguing prospect is whether chronic stimulation of Opto-mGluR6 in bipolar cells may prevent the degenerative process of remodeling within the inner *rd1* retina and potentially protect the retina from continuing damage. It is believed that retinal remodeling is caused by deafferentiation due to lacking activity [66]; thus, timely restoration of bipolar cell light sensitivity during the photoreceptor degenerative process may limit maladaptive remodeling.

Nonpathogenic rAAVs possessing low cytotoxicity and immunogenicity [67] and the ability to mediate lifelong transgene expression are currently the most popular vectors used for retinal therapeutic gene delivery in animal models and human clinical trials [68]. Human retinal gene replacement therapy with rAAVs has received investigational new drug status and has been successfully tested in human patients without significant toxicity or immunogenic reactions [69,70]. This makes rAAV vectors ideal for introducing optogenetic tools stably and robustly into specific retinal neurons [7,8,9,12]. However, wild-type rAAV capsids have a negligible efficacy for bipolar cell transduction. This may have several reasons: (1) the sandwich position of bipolar cells in the retina, including the tight barrier formed by the inner limiting membrane, which may hinder rAAVs to reach bipolar cells in great number, (2) the ability of rAAV capsids to bind onto bipolar cells, and (3) suboptimal intracellular rAAV trafficking and capsid breakdown. In this study, we used capsid-engineered rAAVs with tyrosine-to-phenylalanine mutated residues [30], which were able to infect sufficient numbers of bipolar cells to induce RGC light responses. However, light-triggered responses were not recovered in all RGCs, response latencies and variability were increased, and the maximum RGC spike rates decreased to one-third of the spike rates measured in RGCs of transgenic *rd1*_Opto-mGluR6 mice. An increased viral-mediated expression is therefore imperative for potential future clinical applications. Two recent reports introduced modified rAAV capsid variants that showed a ~7-fold increased ON-bipolar transduction efficiency of ChR2 when compared to the tyrosine-to-phenylalanine mutant rAAV capsids used in this study [53,54]. Whilst previous studies with low ChR2 expression only recovered ON responses in RGCs [9,12], the use of the new rAAV variants led to the recovery of ON and OFF RGC responses, confirming that a strong ON-bipolar cell drive is imperative to indirectly activate OFF-bipolar cells via the AII amacrine cell network.

Aside from its therapeutic potential, Opto-mGluR6 provides a novel approach to studying bipolar cells and inner retinal circuits. Moreover, knowledge of engineering light-activatable, bleach-resistant mGluRs will facilitate investigations into the roles of mGluRs in synaptic plasticity, memory, and disease.

## Materials and Methods

### Animals

Only data from mice aged >p168 were included in this study, at which point retinal light responses were no longer detected in the *rd1* lines (S3 Fig).

Animal experiments and procedures were in accordance with the Swiss Federal Animal Protection Act and approved by the animal research committee of Bern (approval number BE44-12) and by the Niedersächsisches Landesamt für Verbraucherschutz und Lebensmittelsicherheit (registration number 33.9-42502-04-12/0868). Animals were maintained under a standard 12-h light-dark cycle, under enhanced lighting provided with a Philips HF3319 daylight lamp (10,000 lux) positioned 10 cm from the cage to guarantee sufficient light levels for Opto-mGluR6 activity.

#### a) Mouse lines

We purchased four mouse lines from Charles River Laboratories, the European distributor of The Jackson Laboratory, which maintains its own FVB/N strain (FVB/NCrl). (1) The C3H/HeOuJ and (2) FVB/NCrl mouse lines, both homozygous for the Pde6b^rd1^ mutation and suffering from early onset photoreceptor degeneration [71]. In this manuscript, we refer to both mouse lines as *rd1* mice. (3) The C3Sn.BLiA-*Pde6b* ^+^/DnJ and (4) FVB.129P2-*Pde6b* ^+^ Tyr^c-ch^/AntJ mouse lines are congenic to the first two mouse lines, with the wild-type PDE6B allele bred into the corresponding genetic background to offer the characteristics of the C3H and FVB lines without retinal degeneration. The C3Sn and FVB.129P2 mouse lines were crossed and F1 mice used as positive, seeing controls and referred to as *Pde6b* ^+^ in the manuscript.

We generated a FVB/N_Opto-mGluR6 knock-in founder line (see Molecular Biology section), which expresses Opto-mGluR6-IRES-Turbo635 in all of its ON-bipolar cells.

To generate pigmented, blind (*rd1*), and transgenic *rd1*_Opto-mGluR6 mice, we crossbred the FVB/N_Opto-mGluR6 line with C_3_H/HeOu mice. Only F1 mice were used in the experiments, and transgenic offspring were identified by PCR genotyping using the primers GGA GAG GAT CAC CAC ATA CG (F) and GTC TCC TGT CCA CGA AGT AG (R) that bind to the Turbo FP635 transgene and amplify an expected amplicon of 326 base pairs.

Lastly, a pigmented transgenic but seeing mouse line without the Pde6b^rd1^ mutation but otherwise genetically identical was generated by crossbreeding the sighted C3Sn.BLiA-*Pde6b* ^+^/DnJ mouse line with FVB/N_Opto-mGluR6. This seeing transgene mouse line is referred to as *Pde6b* ^+^_Opto-mGluR6 in the manuscript.

#### b) Ocular virus administration

Mice were anesthetized by intraperitoneal injection of 100 mg/kg ketamine and 10 mg/kg xylazine. The pupil of the right eye was dilated with a drop of 10 mg/ml atropine sulfate (Théa Pharma). We then punctured the sclera on the nasal side ~1 mm from the corneal limbus using a 30G needle. The puncture needle was removed, and a 33G blunt needle was maneuvered through the hole to the desired injection site (RPE injection kit from World Precision Instruments). We then injected 1.0–1.5 μl of rAAV vector solution and waited for 10 s before retracting the injection needle form the eye. Following surgery, the eyelids were stuck closed using petroleum jelly to prevent drying of the cornea. The left eye served as control.

### Molecular Biology

#### a) Cloning of chimeric melanopsin-mGluR6 variants

Melanopsin was amplified from mouse retinal cDNA using a forward primer with an EcoRI overhang (TTC GAA TTC GCC ACC ATG GAC TCT CCT TCAG GAC) and a reverse primer with a XmaI overhang (TAT ACC CGG GCA CAG TCA CAT GCA GAT ATT CCC) and cloned into the multiple cloning site of a pIRES2-EGFP vector (Addgene) that was modified to contain TurboFP635 behind the IRES site (a kind gift from A. Nicke). To create the plasmids of the melanopsin-mGluR6 chimeras (pIRES_melanopsin-mGluR6_TurboFP635), the intracellular loops and C-terminus from mouse mGluR6 were synthesized as primer overhangs and introduced using overlap extension PCR [72]. Only the primers used for generating the final version pIRES_Opto-mGluR6_TurboFP635 are listed here; for other chimeric variants, the overlap primers were adapted accordingly. DNA primers used to exchange IL2 were as follows: CAT CAC TTC CAT GAT CAC CC (F1), CGT GAC AGA GCG CTT CCC TTG CTC GAA AAT GCG GTAG ATG CGG TCC ATG GCT ATG GCT GT (R1), GGG AAG CGC TCT GTC ACG CCG CCA CCC TTC ATC AGC CCC ACC TCG CAG GTC CTG CTA GGC GTC TGG (F2), and CTT CGG CCA GTA ACG TTA GGG (R2). The underlined areas indicate the used restriction sites (F1 and R2) as well as the inserted overhang sequences (R1 and F2). When exchanging IL3 and the C-terminus, we used the same F1 and R2 primers from above, but the overhang primers were CAT TGA AGG TCT CTG GCA CAC CTC GGG CCC TGA AGA TGA AGA TG (R1) and GTG TGC CAG AGA CCT TCA ATG AAG CCA AGG TCG CAC TGA TTG TCA TTC (F2) for IL3 and CCA TCG TGG AGG TCT TCT TGA GGC TGC GCT TCC GCT TCT GCA CGT TCT GCT CGG GGT GAG TGA TGG CGT AGA (R1) and CAA GAA GAC CTC CAC GAT GGC GGC CCC GCC CAA GAG CGA GAA CTC AGA GGA CGC CAA GTA GCC CGG GGA GAG ATG GGA TC (F2) for the C-terminus.

#### b) rAAV generation

The short, ON-bipolar cell—specific mouse *GRM6* enhancer sequence in combination with the sv40 viral promoter was cloned into the pGL3 luciferase reporter vector (Promega) as described previously [29]. Subsequently, the transgene Opto-mGluR6_IRES_TurboFP635 was excised from the pIRES_Opto-mGluR6_TurboFP635 plasmid using the flanking XhoI and XbaI restriction sites and cloned into the pGL3 vector to replace the native luciferase gene and generate pGL3_*GRM6*/sv40_Opto-mGluR6_IRES_TurboFP635. We then PCR-amplified the *GRM6*/sv40_Opto-mGluR6_IRES_TurboFP635 sequence (F: GAA TTC GCC CTT AAG CTAG CGT CGA TCT CCA GAT GGC TAA AC, R: GAT TGG ATC CAA GCT TCA GCT GTG CCC CAG TTT G) and inserted it into a linearized pAAV2.1-Rho-EGFP vector (kind gift from A. Auricchio) [73], using an In-Fusion 2.0 homologous recombination Kit (Clontech). The AAV packaging was carried out at the University Clinic in Cologne (H. Büning, Centre for Molecular Medicine). We used a mutated serotype 2 capsid in which six exposed phenylalanine residues were substituted by tyrosine (Y252, 272, 444, 500, 704, and 730F) [74]. Viral vectors were produced in HEK293 cells by the triple plasmid cotransfection method and empty virions removed by density purification over an iodixanol gradient. The 40% iodixanol fraction was subsequently buffer exchanged by column chromatography over a 5 ml HiTrap heparin affinity column (Sigma-Aldrich). The vector was eluted from the column using 1 M NaCl and the rAAV fraction titered for DNase-resistant vector genomes by real-time PCR relative to a standard vector. The final fraction with a titer of 8 10^12^ genome copies per ml was stored at −80°C.

#### c) Generation of the Opto-mGluR6 transgenic mouse plasmid

We generated Opto-mGluR6-IRES-Turbo635 knock-in mice using the full-length mGluR6 promoter to prevent long-term silencing of the transgene that often occurs when using viral promoters. For this, we amplified the entire pGL3_mGluR6/sv40_Opto-mGluR6_IRES_TurboFP635 vector excluding the mGluR6/sv40 promoter enhancer using the DNA primers TAT AGC TAG CGC CAC CAT GGA CTC TCC TTC AGG ACC A (F) and TAT ACT CGA GAG AAA TGT TCT GGC ACC TGC (R), which include the NheI and XhoI restriction sites (underlined). We then ligated the amplicon to the full-length mGluR6 promoter cut from the pBluescript II KS(+)_PRGRM6 plasmid (a kind gift from R. Duvoisin). The pGL3_mGluR6_Opto-mGluR6_IRES_TurboFP635 plasmid was used to generate transgenic FVB/N mice by standard pronuclear injection (PolyGene, Zurich, Switzerland).

### Immunocytochemistry and Microscopy

Retinas were dissected and fixed in 4% paraformaldehyde in 0.1 M phosphate buffer (pH 7.4) for 30 min. For frozen sections, retinas were cryoprotected in graded sucrose solutions before freezing in OCT medium (Sakura Finetek). 15-μm-thick cryosections were mounted on SuperFrost slides (Menzel). Antibodies were diluted in a blocking solution containing 1% Triton-X and 2% donkey serum. Cryosections were incubated overnight at 4°C in primary antibody and 2 h in secondary antibody at room temperature, while whole-mount retinas were incubated for 5 d in primary and 2 h in secondary antibody at 4°C. The following primary antibodies were used: rabbit anti-TurboFP635 (1:1000; Evrogen; AB234), goat anti-melanopsin (1:1000; Advanced Targeting Systems; AB-N38), mouse anti-PKCα (1:1000; Santa Cruz; sc-8393), goat anti-ChAT (1:100; Millipore; AB144P), mouse anti-Goα (1:1000; Chemicon; MAB3073), goat anti—OPN1 SW antibody (1:500, Santa Cruz, sc-14365), mouse anti-CD45 (1:500; eBiosciences; 12–0460), and rabbit anti-GFAP (1:500; Dako; Z0334). Secondary antibodies were always from donkey and either conjugated to Alexa 488 or Alexa 594 (1:400; Invitrogen). Alexa 488 conjugated to streptavidin was used to visualize cells injected with neurobiotin during patch-clamp experiments (1:400; Invitrogen; S-11223). Nuclei were stained with 10 μg/ml DAPI (Roche). Micrographs were taken on a Zeiss Laser Scanning Microscope 510. Processing of image stacks was done using ImageJ (Rasband WS, United States National Institutes of Health, Bethesda, Maryland, US).

### Electrophysiological Recordings

#### a) Whole-cell voltage clamp on HEK293-GIRK cells

HEK293 cells stably expressing Kir3.1/3.2 potassium channels (HEK293-GIRK; a kind gift from L. Jan) [75] were transfected with pIRES_Opto-mGluR6_TurboFP635 using Lipofectamine 2000 (Invitrogen) and the culture medium supplemented with 0.25 μM 9-cis and 0.5 μM all-trans retinal to reconstitute melanopsin. Transfected HEK293-GIRK cells were patched after 24–48 hrs. The bath solution contained 25 mM KCl, 102 mM NaCl, 1mM MgCl2, 25 mM Hepes buffer, and 30 mM D-Glucose (pH 7.4). The pipette solution contained 129 mM K-Gluconate, 10 mM KCl, 10 mM Hepes, 4 mM MgATP and 0.3 mM Na_3_GTP (pH 7.3). Patch pipettes with resistances of 2–4 MΩ were pulled from borosilicate glass (GB150-8P, Science Products) on a horizontal DMZ-Universal puller (5318904120B, Zeitz Instruments). Opto-mGluR6 was activated by light pulses from a diode-pumped solid-state laser (Pusch Opto Tech GmbH, λ = 473 nm) focused into a 400-μm optic fiber and controlled by a fast computer-controlled shutter (Uniblitz LS6ZM2, Vincent Associates). We used blue light since Opto-mGluR6 contains the unaltered light switch of mouse melanopsin that was shown to respond maximally to 467 nm [17]. The light intensity at the end of the light guide was ~10^18^ photons cm^-2^ s^-1^. Current-voltage curves in darkness and under illumination were generated between -150 mV and +70 mV in 10 mV steps and the differential current at -150 mV calculated to determine the light-activated GIRK conductance. All HEK293-GIRK experiments were performed at room temperature.

#### b) Cell-attached recordings from retinal ganglion cells

The methods for recoding cell-attached light responses from RGCs have been described in detail previously [60]. Voltage recordings were made using a HEKA EPC9 amplifier with Pulse software. Mice were euthanized, and the retinas were dissected out and placed in a recording chamber perfused with Ames medium (Sigma-Aldrich) at 34–36°C. Electrodes were pulled from borosilicate glass to a final resistance of 5–8 MΩ and filled with Ames medium. RGCs were targeted and approached under visual control using infrared differential-interference-contrast (IR-DIC) optics until spontaneous action potentials were observed in the voltage recording. Blue-light (λ = 465 nm, 5.4 x 10^16^ photons s^-1^ cm^-2^) flash stimuli were generated by a pE-2 system (CoolLED, Andover, United Kingdom) and projected through a 40 water immersion objective onto the receptive field of the recorded RGC. The stimulus period was triggered directly by the Pulse software. Stimulus intensity was controlled using neutral density filters in the light path. The background light intensity was kept near zero. Traces were analyzed offline using Igor Pro software (Wave Metrics) and a series of routines written by W. R. Taylor.

#### c) Whole-cell patch-clamp recordings from bipolar cells

Bipolar cell patching was performed as described previously [76]. After dissection, dark-adapted retinas were imbedded in 1% low-melting agar in HEPES buffered Ames medium at 40°C and immediately cooled on an ice block to solidify the agar. Retinal sections with a thickness of 200 μm were cut using a Camden instruments vibratome and placed in a recording chamber perfused with Ames medium (Sigma-Aldrich) at 34–36°C. Patch electrodes were pulled from borosilicate glass to a final resistance of 6–8 MΩ and filled with an intracellular solution containing (in mM) KCL 110, NaCl 10, MgCl_2_ 1, EGTA 5, CaCl_2_ 0.5, HEPES 10, GTP 1, cGMP 0.1, ATP 1, and cAMP 0.05 (pH adjusted to 7.2 with KOH). We recorded successfully from 14 rod bipolar cells and seven cone bipolar cells (two OFF and five ON). Bipolar cells were characterized upon their response kinetics [41]. Rod bipolar cells had sluggish sustained responses to long stimuli (2 s) that were clearly shortened in response to brief stimuli (50 ms).

#### d) Elimination of the photoreceptor input: Photopigment bleaching and pharmacological block

Photoreceptor bleaching in *Pde6b* ^+^_Opto-mGluR6 mice was accomplished with maximal epifluorescence illumination with a 150 W Xenon lamp (5.35 x 10^17^ photons s^-1^ cm^-2^) for 5 min. This protocol leads to complete bleaching of both rods and cones, as calculated by the equation *F = 1-exp(-IPt)* [40], with *F* the fraction of bleached pigment, *I* the light intensity (5.3 x 10^17^ photons cm^-2^ s^-1^), *t* the time of light exposure (300 s), and *P* the photoreceptor’s photosensitivity (6.0 x 10^-9^ μm^-2^ for cones [77] and 6.2 x 10^-9^ μm^-2^ for rods [78]). Although photoreceptors in isolated retinas are unable to recover from bleaching, we additionally added 20 μM L-AP4 to the bath solution for direct bipolar cell recordings to guarantee elimination of residual photoreceptor input. To simulate “light-adapted,” natural viewing conditions for Opto-mGluR6 recordings, we also turned the room lighting on after bleaching.

#### e) Electroretinography

All procedures were performed under dim red illumination. We anesthetized mice with an intraperitoneal injection of 100 mg/kg ketamine and 10 mg/kg xylazine and dilated the pupils of both eyes using a drop of 10 mg/ml atropine sulfate (Théa Pharma). Animals were subsequently placed on a temperature-controlled heating table inside a Q400 full-field bowl (Roland Consult, Brandenburg, Germany). We recorded from both eyes using gold wire electrodes wetted with 1% methylcellulose that circled the corneal borders (OmniVision). Needle electrodes placed under the skin of the cheeks were used as reference electrodes, while a third needle electrode positioned in the base of the tail served as a ground electrode. Responses to strobe flashes were amplified, averaged (average of 20 consecutive recordings), and stored using a RetiScan-RetiPort electrophysiology unit (Roland Consult). Pharmacology was performed by intravitreal injection of 1 μl synaptic blocker mix into the right eye only (the left eye served as internal control). The mix comprised either L-AP4 (a specific agonist of glutamate metabotropic receptors) with DL-APV (an NMDA receptor antagonist) or the two substances together with CNQX (an AMPA/Kainate receptor antagonist). Assuming a vitreal volume of approximately 25 μl of a mouse eye, final intravitreal concentrations were 250 μM of DL-APV, 1 μM of L-AP4, and 40 μM of CNQX.

### Intrinsic Signal Optical Imaging of V1-Activity

#### a) Surgery

Mice were initially box-anaesthetized with 2% halothane in a mixture of O_2_:N_2_O (1:1) and received atropine (Franz Köhler, 0.3 mg/mouse, subcutaneously), dexamethasone (Ratiopharm, 0.2 mg/mouse, subcutaneously), and chlorprothixene (Sigma, 0.2 mg/mouse, intramuscularly). Mice were placed in a stereotaxic frame, and anaesthesia was maintained with 0.8%–1.2% halothane in a 1:1 mixture of O_2_:N_2_O applied through a tube attached to the nose. Body temperature was maintained at 37°C, and the heart rate was monitored throughout the experiment. Lidocaine (2% xylocaine jelly) was applied locally to all incisions. The skin above the skull was incised to expose V1 of both hemispheres, and agarose (2.5% in 0.9% NaCl) and a glass coverslip were placed over the exposed area.

#### b) Optical imaging

Visual cortical responses of the left and right hemisphere were imaged through the intact skull using the method developed by Kalatsky and Stryker [48]. In this method, a temporally periodic stimulus is continuously presented to the animal, and the cortical responses at the stimulus frequency are extracted by Fourier analysis. Optical images of blood oxygenation were recorded using a CCD (Dalsa) camera with a 135 mm x 50 mm tandem lens configuration (Nikon) controlled by custom software. The surface vascular pattern and intrinsic signal images were visualized with illumination wavelengths set by a green (550 ± 10 nm) or red (610 ± 10 nm) interference filter, respectively. After acquisition of a surface image, the camera was focused 600 μm below the cortical surface. An additional red filter was interposed between the brain and the CCD camera. Frames were acquired at a rate of 30 Hz temporally binned to 7.5 Hz and stored as 512 x 512 pixel images after spatial binning of the camera image. A cortical area of 4.6 × 4.6 mm^2^ was imaged for both left and right visual cortex.

#### c) Visual stimuli

Visual stimuli consisted of moving horizontal bars (4° wide, temporal frequency 0.125 Hz) extending over 78° in vertical extent and over 129° in horizontal extent, generated by a Matrox G450 board (Matrox Graphics, Quebec, Canada), controlled by custom software. To ensure sufficiently high-stimulus light-intensities for Opto-mGluR6 activation, stimuli were intensity-amplified to a luminance of 3800 lux on a 20-lux background using a multimedia projector (Seiko Epson Corporation, lamp type ELPLP67) projecting onto a transparent rear-projection screen placed at a distance of 25 cm in front of the animal (light intensity at the position of the mouse was ~240 candela). Stimuli were presented binocularly and positioned in the left visual field for imaging the right hemisphere (-83° to 46° azimuth) and to the right visual field for imaging the left hemisphere (-46° to 83° azimuth).

#### d) Data analysis

Visual cortical maps were calculated from the acquired frames by Fourier analysis to extract the signal at the stimulation frequency using custom software [48]. While the phase component of the signal is used for the calculation of retinotopy (color coded), the amplitude component represents the intensity of neuronal activation (expressed as fractional change in reflectance ×10^-4^) and was used to calculate V1 activation [79]. In polar maps, hue encodes visual field position (retinotopy), and lightness encodes the magnitude of the visual responses. Two-to-three maps from both hemispheres of one animal were averaged for further quantification and data display using Matlab. For the comparison of V1 activation between transgenic and nontransgenic *rd1* mice, we pooled averaged values from individual hemispheres of both genotypes.

### Behavioral Experiments

#### a) Optomotor reflex

The OptoMotry system from CerebralMechanics (Lethbridge, Alberta, Canada) was used to measure the OKR with the method described previously [47,80]. A virtual cylinder comprising a vertical sine wave grating was projected in 3-D coordinate space on four computer monitors (Dell 1703FP) facing a 13-cm-high platform above a mirrored floor under a likewise mirrored lid. The light intensity measured at the centre of the platform was 5.6 10^13^ photons cm^-2^ s^-1^, sufficient to activate Opto-mGluR6. During testing, the mice stood unrestrained on the platform, tracking the 3-D pattern with a reflexive head movement that was recorded with a video camera (DCR-HC26, Sony). The movements of the mice on the platform were followed by two independent experimenters with a crosshair superimposed on the video image to center the rotation of the virtual cylinder with the x-y positional coordinates of the crosshair at the head of the mouse. The spatial frequency of the test grating was changed in steps until the highest spatial tracking frequency was identified as the threshold (visual acuity).

#### b) Water maze experiments

We used a water maze system commonly used to assess sensory abilities in rodents [80]. The water maze consisted of a circular pool (1.8 m in diameter, 0.7 m in height) filled with opaque water (23°C) dyed with white nontoxic emulsion paint (Bahag AG). A submerged platform (20-cm diameter) with a centrally mounted LED tower that carried a ring of six evenly spaced LEDs (460 nm, ASMT-JB31-NMP01) ~5 cm above the water surface was placed in one quadrant of the tank. The maximum light power at the furthest location in the pool was ~110^16^ photons cm^-2^ s^-1^. Prior to training, mice were released 10 cm in front of the platform, facing the platform, and allowed to exit the water onto the platform, where they were left for 30 s before they were returned to their cages. For the swim task, the mice were randomly released at one of three entry points and left for 3 min to find the platform. If a mouse found the platform, it was allowed to rest for 30 s before it was returned to its cage. Mice that failed to reach the platform were guided manually onto the platform and also left for 30 s. Experiments were recorded with a high-resolution camera (CCD-IRIA, Sony) placed above the pool and connected to a computer running Ethovision software (Noldus Information Technology, Wageningen, The Netherlands). Releasing a mouse in one of the specified release sites triggered an automatic recording of the swim path. Each mouse swam three trials per day during the training period in which the averaged distance swam over the three trials was used as a measure of daily performance. It is important to note that light intensity was not static in these experiments and that the given intensities were measured at the furthest location in the pool, as stated in the text.

### Statistical Analysis

Statistics were performed either by Excel or NCSS statistics software. For all normal distributions (verified using the Kolmogorov-Smirnov test), differences between Opto-mGluR6 and control groups were analyzed using an unpaired one-tailed Student’s *t* test not assuming equal variance. As a result of the experimental cutoff time (3 min) and individual learning, swim distances in the water maze trials were not expected to have a normal distribution. In these experiments, significance levels were determined using a Mann-Whitney 'U' test. In the figures, different levels of significance are indicated by * if *p* < 0.05, ** if *p* < 0.01, and *** if *p* < 0.001. In the text, average values are indicated ± standard deviation, unless otherwise indicated.

## Supporting Information

S1 Data Excel spreadsheet containing, in separate sheets, the underlying numerical data and statistical analysis for Figs 4C, 8B, 9A, 9B, and 9C, S3 Fig, and S1 Table. (XLSX)

S2 Data Plasmid maps and DNA sequences of Melanopsin-mGluR6 chimeras and promoters. (DOCX)

S1 Fig Expression under the short *GRM6*/sv40 enhancer promoter sequence is not ON-bipolar cell specific. Immunolabeling against Gαo (red) and the Opto-mGluR6 reporter gene TurboFP635 (green) of an rAAV-*GRM6*/sv40-Opto-mGluR6-TurboFP635 transduced *rd1* retina clearly indicates, in addition to ON-bipolar cell labeling (solid arrow), nonspecific labeling of amacrine and RGCs (open arrows). Scale bar, 50 μm.
(TIF)

S2 Fig Evaluation of potential immunogenicity and cytotoxicity of Opto-mGluR6 in retinal cryosections. CD45 (red), a general marker for immune cells, and GFAP (green), an astrocyte marker, were used to label retinas of FVB/N mice (A) intravitreally injected with pAAV2.2(Y252,272,444,500,704,730F)-*GRM6*/sv40_Opto-mGluR6_IRES_ TurboFP635 and (B) *rd1* littermates 8 mo after virus injection. As exemplified in the above confocal micrographs, we observed no CD45 expression in injected and noninjected eyes indicating limited presence of immune cells (red areas indicate nonspecific blood vessel labeling of secondary antimouse antibody). We also observed no change in the number of Müller glia cells (green), known to increase upon retinal injury. Nuclei are labeled by DAPI (blue). Scale bars, 20 μm.
(TIF)

S3 Fig Fraction of responding RGCs versus age in the two *rd1* strains used, FVB/N (for rAAV injections) and FVB/NxC3H/HeOu (transgenic animals). During cell attached recordings, a 1-s light step stimulus (5.4 x 10^16^ photons s^-1^ cm^-2^) was projected over the receptive field of the RGC. The stimulus was presented five times to evaluate if action potentials were modulated in a consistent manner. To be classed as light sensitive, spike-time histograms (100-ms bins) generated from all five traces had to have a significant change in firing frequency between at least two bins generated before and after the onset of the light stimulus. In both strains, the fraction of RGCs responding to the light stimulus decreased sharply with age, with a faster attenuation in FVB mice, which had no RGC responses by 12 wk of age compared to C3H/FVB mice, which lost RGC light responses by 24 wk.
(TIF)

S4 Fig Example light intensity response traces used to generate Fig 4C. Extracellular recordings from an ON-RGC in a transgenic *rd1*_Opto-mGluR6 retina. Light flashes of various intensities were presented for 1 sec from 0.5–1.5 sec (between the broken vertical lines). The cell starts responding at a light intensity of 5 x 10^11^ photons cm^-2^ s^-1^. As the light intensity increases, the response strengthens. To generate the light intensity response curve depicted in Fig 4C, the spikes for each RGC were counted over the period of the light stimulus using a threshold detection routine written in IgorPro software. For cells with baseline activity, as in the given example, a corresponding trace with no light flash was recorded and the given spike count subtracted from all counts recorded during light stimulation. The spike count was subsequently scaled to a maximum of 1 and plotted against a logarithmic light intensity scale. Average maximum spike counts per light pulse: transgenic retina, 49 ± 18, rAAV-transduced retina, 15 ± 3 spikes (mean ± SEM). Note that the response latency increases with decreasing light intensity.
(TIF)

S5 Fig Illustration of the sign inversion of the light signal when ON-bipolar cells are activated through Opto-mGluR6. There are two classes of bipolar cells in the retina, ON bipolars and OFF bipolars, which provide parallel information channels responding to increases versus decreases in light intensity. Whilst cone bipolar cells (receiving input from cones) are divided into ON and OFF type (ON CB and OFF CB), rod bipolar cells (RB), receiving input from rods, are all of the ON type. Since rod bipolar cells are by far the most numerous ON-bipolar cells, the majority of Opto-mGluR6 expressing cells will be rod bipolar cells [81]. (A) illustrates the intact rod pathway: upon illumination, the rod bipolar cell (RB, orange) is depolarized, which subsequently depolarizes the AII amacrine cell (blue). In turn the cone ON-bipolar cell (ON CB, green) is also depolarized through a sign-conserving electrical synapse, while the cone OFF-bipolar cell (OFF CB, red) is hyperpolarized through a sign-inverting glycinergic synapse. Finally, ON ganglion cells (GC, green) are depolarized and OFF ganglion cells (GC, red) are hyperpolarized by light. (B) illustrates the degenerated pathway recovered through Opto-mGluR6 (red dots) expressed in ON-bipolar cells: upon illumination, rod bipolar cells and subsequently AII amacrine cells are hyperpolarized. The cone ON-bipolar cells are also hyperpolarized, directly by light and indirectly by the AII amacrine cells, and the cone OFF-bipolar cells are depolarized through the sign-inverting gap junction from AII cells. As a consequence, native ON-RGCs (green) are now hyperpolarized (now OFF-RGCs), and native OFF-RGCs (red) are now depolarized (now ON-RGCs). Black arrows indicate how rod bipolar cells feed into the native OFF circuitry.
(TIF)

S6 Fig Cone opsin and melanopsin staining in *rd1* retinas. (A,B) Micrographs were taken from the ventral periphery of six OPN1SW-labeled *rd1* retinas, three at p180 and three at p280, and the density of labeled photoreceptors counted. Counts were 329 ± 89 cells/mm^2^ for p180 retinas (example shown in A) and 231 ± 41 cells/mm^2^ for p280 retinas (example shown in B). Surviving cones expressed opsin in their somas and typically grew long dendritic processes (empty arrows). They had no cilia, although sparse round opsin-labeled structures may represent remnant outer segments (solid arrows). (C,D) *Rd1* retinas at p180 (C) and p280 (D) were labeled for opn4. Micrographs were taken in the midperiphery of three retinas from each age group and the number of melanopsin-labeled cell bodies counted. Counts were 22 ± 5 for p180 retinas and 19 ± 3 for p280 retinas. Scale bar is 50 μm.
(TIF)

S1 Table GIRK conductances measured in HEK293-GIRK cells expressing the melanopsin-mGluR6 variants. Although all constructed chimeras were functional and activated not significantly different GIRK conductances, CM III showed the highest mean activity out of the variants CM I-CM VII and was therefore further modified (bold typing). The improved CM III_ΔL variant (boxed) is in this manuscript referred to as Opto-mGluR6.
(TIF)

S1 Text (A) Overview of amino acid (AA) sequences of the different melanopsin-mGluR6 variants and terminology. (B) Amino acid sequences of all constructed and functionally tested chimeras.
(DOCX)

## Abbreviations

AMD: age-related macular degeneration
BGHpA: bovine growth hormone polyadenylation sequence
ChR2: Channelrhodopsin-2
CNQX: 6-cyano-7-nitroquinoxaline-2,3-dione
CPD: cycles per degree
DL-APV: DL-2-Amino-5-phosphonopentanoic acid
ERG: electroretinogram
GIRK: G-protein coupled inward rectifying potassium channel
GPCR: G-protein-coupled receptor
*GRM6*: mGluR6 enhancer element
HEK293 cell: human embryonic kidney cell
IL: intracellular loop
IPL: inner plexiform layer
ipRGC: intrinsically photosensitive retinal ganglion cell
IR-DIC: infrared differential interference contrast
IRES: internal ribosomal entry site
ITR: internal tandem repeat
L-AP4: L-(+)-2-amino-4-phosphonobutyric acid
LED: light-emitting diode
miRNA: microRNA
NMDA: *N*-Methyl-D-aspartic acid
OKR: optokinetic reflex
ON-BP: ON-bipolar cell
psv40: sv40 eukaryotic promoter sequence
rAAV: recombinant adeno-associated virus
RGC: retinal ganglion cell
RGS: regulators of G-protein signaling proteins
RP: retinitis pigmentosa
SEM: standard error of the mean
TM: transmembrane
V1: primary visual cortex
WPRE: woodchuck posttranscriptional regulatory element
