# Supplementary material for: Restoring the ON Switch in Blind Retinas: Opto-mGluR6, a Next-Generation, Cell-Tailored Optogenetic Tool
Source: PLoS Biol. 2015 May 7;13(5):e1002143. doi: 10.1371/journal.pbio.1002143 (PMC4423780; doi:10.1371/journal.pbio.1002143)
Supplement: S2 Data — (DOCX) [file pbio.1002143.s002.docx]

**Plasmid maps and DNA sequences**

1. **Plasmid maps**

(1) pIRES2-CMV/CAG-Opto(CMI-CMVII)_mGluR6_TurboFP635

Plasmid for HEK293-GIRK cell transfections

(2) pGL3_PRmGluR6_Opto-mGluR6_IRES_TurboFP635

Plasmid for pronuclear injections


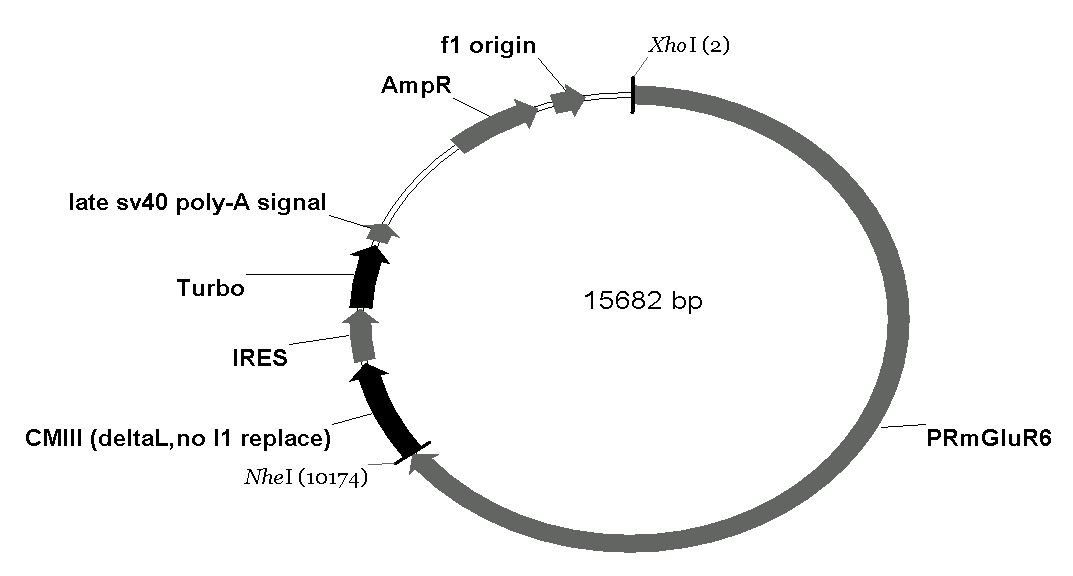


(3) pAAV2_*GRM6*/sv40_Opto-mGluR6_IRES_TurboFP635

Plasmid for rAAV packaging


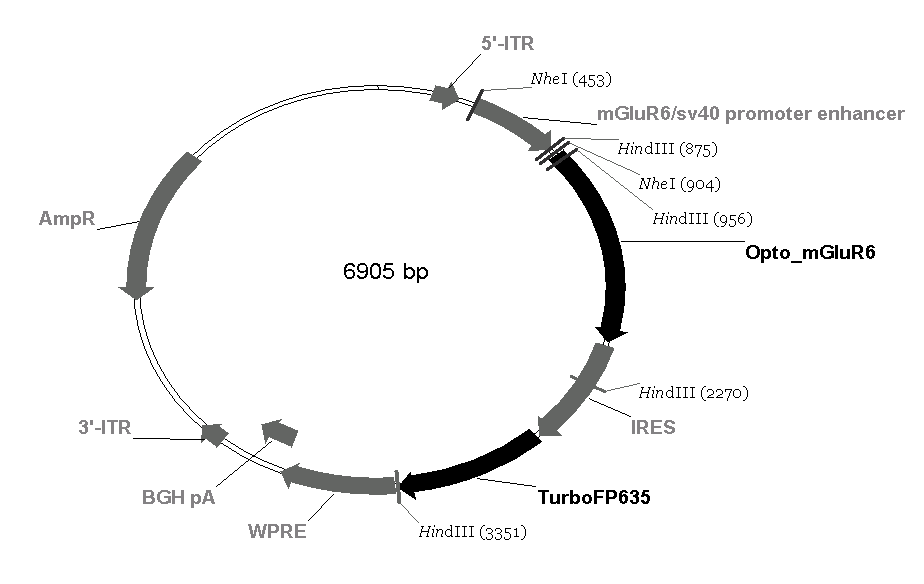


1. **DNA sequences**

*Melanopsin_mGluR6 chimeras*

1. CMI (GenBank accession number KR005385)

ATGGACTCTCCTTCAGGACCAAGAGTCTTGTCAAGCTTAACTCAGGATCCCAGCTTCACAACCAGTCCTGCCCTGCAAGGCATTTGGAACGGCACTCAGAACGTCTCCGTAAGAGCCCAGCTTCTCTCTGTTAGCCCCACGACATCTGCACATCAGGCTGCTGCCTGGGTCCCCTTCCCCACAGTCGATGTCCCAGACCATGCTCACTATACCCTAGGCACGGTGATCCTGCTGGTGGGACTCACAGGGATGCTGGGCAATCTGACGGTCATCTACACCTTCTGCAGGAACAGAGGCCTGCGGACACCAGCAAACATGTTCATCATCAACCTCGCAGTCAGCGACTTCCTCATGTCAGTCACTCAGGCCCCGGTCTTCTTTGCCAGCAGCCTCTACAAGAAGTGGCTCTTTGGGGAGACAGGTTGCGAGTTCTATGCCTTCTGCGGGGCTGTCTTTGGCATCACTTCCATGATCACCCTGACAGCCATAGCCATGAAGACCAACCGCATCTACCGCATTTTCGAGCAAGGGAAGCGCTCTGTCACGCCGCCACCCTTCATCAGCCCCACCTCGCAGCTCGTCCTGCTAGGCGTCTGGCTTTATGCCCTGGCCTGGAGTCTGCCACCTTTCTTTGGTTGGAGTGCCTACGTGCCCGAGGGGCTGCTGACATCCTGCTCCTGGGACTACATGACCTTCACACCCCAGGTGCGTGCCTACACCATGCTGCTCTTCTGCTTTGTCTTCTTCCTCCCCCTGCTCATCATCATCTTCTGCTACATCTTCATCTTCAGGGCCCGAGGTGTGCCAGAGACCTTCAATGAAGCCAAGGTCGCACTGATTGTCATTCTTCTCTTCGTGCTGTCCTGGGCTCCCTACTCCACTGTGGCTCTGGTGGCCTTTGCTGGATACTCGCACATCCTGACGCCCTACATGAGCTCGGTGCCAGCCGTCATCGCCAAGGCTTCTGCCATCCACAATCCCATTATCTACGCCATCACTCACCCCGAGCAGAACGTGCAGAAGCGGAAGCGCAGCCTCAAGAAGACCTCCACGATGGCGGCCCCGCCCAAGAGCGAGAACTCAGAGGACGCCAAGTAG

1. CMII (GenBank accession number KR005386)

ATGGACTCTCCTTCAGGACCAAGAGTCTTGTCAAGCTTAACTCAGGATCCCAGCTTCACAACCAGTCCTGCCCTGCAAGGCATTTGGAACGGCACTCAGAACGTCTCCGTAAGAGCCCAGCTTCTCTCTGTTAGCCCCACGACATCTGCACATCAGGCTGCTGCCTGGGTCCCCTTCCCCACAGTCGATGTCCCAGACCATGCTCACTATACCCTAGGCACGGTGATCCTGCTGGTGGGACTCACAGGGATGCTGGGCAATCTGACGGTCATCTACACCTTCTGCAGGAACAGAGGCCTGCGGACACCAGCAAACATGTTCATCATCAACCTCGCAGTCAGCGACTTCCTCATGTCAGTCACTCAGGCCCCGGTCTTCTTTGCCAGCAGCCTCTACAAGAAGTGGCTCTTTGGGGAGACAGGTTGCGAGTTCTATGCCTTCTGCGGGGCTGTCTTTGGCATCACTTCCATGATCACCCTGACAGCCATAGCCATGAAGACCAACCGCATCTACCGCATTTTCGAGCAAGGGAAGCGCTCTGTCACGCCGCCACCCTTCATCAGCCCCACCTCGCAGCTCGTCCTGCTAGGCGTCTGGCTTTATGCCCTGGCCTGGAGTCTGCCACCTTTCTTTGGTTGGAGTGCCTACGTGCCCGAGGGGCTGCTGACATCCTGCTCCTGGGACTACATGACCTTCACACCCCAGGTGCGTGCCTACACCATGCTGCTCTTCTGCTTTGTCTTCTTCCTCCCCCTGCTCATCATCATCTTCTGCTACATCTTCATCTTCAGGGCCATCAAGGCCCGAGGTGTGCCAGAGACCTTCAATGAAGCCAAGGTCGCACTGATTGTCATTCTTCTCTTCGTGCTGTCCTGGGCTCCCTACTCCACTGTGGCTCTGGTGGCCTTTGCTGGATACTCGCACATCCTGACGCCCTACATGAGCTCGGTGCCAGCCGTCATCGCCAAGGCTTCTGCCATCCACAATCCCATTATCTACGCCATCACTCACCCCGAGCAGAACGTGCAGAAGCGGAAGCGCAGCCTCAAGAAGACCTCCACGATGGCGGCCCCGCCCAAGAGCGAGAACTCAGAGGACGCCAAGTAG

1. CMIII (GenBank accession number KR005387)

ATGGACTCTCCTTCAGGACCAAGAGTCTTGTCAAGCTTAACTCAGGATCCCAGCTTCACAACCAGTCCTGCCCTGCAAGGCATTTGGAACGGCACTCAGAACGTCTCCGTAAGAGCCCAGCTTCTCTCTGTTAGCCCCACGACATCTGCACATCAGGCTGCTGCCTGGGTCCCCTTCCCCACAGTCGATGTCCCAGACCATGCTCACTATACCCTAGGCACGGTGATCCTGCTGGTGGGACTCACAGGGATGCTGGGCAATCTGACGGTCATCTACACCTTCTGCAGGAACAGAGGCCTGCGGACACCAGCAAACATGTTCATCATCAACCTCGCAGTCAGCGACTTCCTCATGTCAGTCACTCAGGCCCCGGTCTTCTTTGCCAGCAGCCTCTACAAGAAGTGGCTCTTTGGGGAGACAGGTTGCGAGTTCTATGCCTTCTGCGGGGCTGTCTTTGGCATCACTTCCATGATCACCCTGACAGCCATAGCCATGGACCGCATCTACCGCATTTTCGAGCAAGGGAAGCGCTCTGTCACGCCGCCACCCTTCATCAGCCCCACCTCGCAGCTCGTCCTGCTAGGCGTCTGGCTTTATGCCCTGGCCTGGAGTCTGCCACCTTTCTTTGGTTGGAGTGCCTACGTGCCCGAGGGGCTGCTGACATCCTGCTCCTGGGACTACATGACCTTCACACCCCAGGTGCGTGCCTACACCATGCTGCTCTTCTGCTTTGTCTTCTTCCTCCCCCTGCTCATCATCATCTTCTGCTACATCTTCATCTTCAGGGCCCGAGGTGTGCCAGAGACCTTCAATGAAGCCAAGGTCGCACTGATTGTCATTCTTCTCTTCGTGCTGTCCTGGGCTCCCTACTCCACTGTGGCTCTGGTGGCCTTTGCTGGATACTCGCACATCCTGACGCCCTACATGAGCTCGGTGCCAGCCGTCATCGCCAAGGCTTCTGCCATCCACAATCCCATTATCTACGCCATCACTCACCCCGAGCAGAACGTGCAGAAGCGGAAGCGCAGCCTCAAGAAGACCTCCACGATGGCGGCCCCGCCCAAGAGCGAGAACTCAGAGGACGCCAAGTAG

1. CMIV (GenBank accession number KR005388)

ATGGACTCTCCTTCAGGACCAAGAGTCTTGTCAAGCTTAACTCAGGATCCCAGCTTCACAACCAGTCCTGCCCTGCAAGGCATTTGGAACGGCACTCAGAACGTCTCCGTAAGAGCCCAGCTTCTCTCTGTTAGCCCCACGACATCTGCACATCAGGCTGCTGCCTGGGTCCCCTTCCCCACAGTCGATGTCCCAGACCATGCTCACTATACCCTAGGCACGGTGATCCTGCTGGTGGGACTCACAGGGATGCTGGGCAATCTGACGGTCATCTACACCTTCTGCAGGAACAGAGGCCTGCGGACACCAGCAAACATGTTCATCATCAACCTCGCAGTCAGCGACTTCCTCATGTCAGTCACTCAGGCCCCGGTCTTCTTTGCCAGCAGCCTCTACAAGAAGTGGCTCTTTGGGGAGACAGGTTGCGAGTTCTATGCCTTCTGCGGGGCTGTCTTTGGCATCACTTCCATGATCACCCTGACAGCCATAGCCATGGACCGCATCTACCGCATTTTCGAGCAAGGGAAGCGCTCTGTCACGCCGCCACCCTTCATCAGCCCCACCTCGCAGCTCGTCCTGCTAGGCGTCTGGCTTTATGCCCTGGCCTGGAGTCTGCCACCTTTCTTTGGTTGGAGTGCCTACGTGCCCGAGGGGCTGCTGACATCCTGCTCCTGGGACTACATGACCTTCACACCCCAGGTGCGTGCCTACACCATGCTGCTCTTCTGCTTTGTCTTCTTCCTCCCCCTGCTCATCATCATCTTCTGCTACATCTTCATCTTCAGGGCCATCAAGGCCCGAGGTGTGCCAGAGACCTTCAATGAAGCCAAGGTCGCACTGATTGTCATTCTTCTCTTCGTGCTGTCCTGGGCTCCCTACTCCACTGTGGCTCTGGTGGCCTTTGCTGGATACTCGCACATCCTGACGCCCTACATGAGCTCGGTGCCAGCCGTCATCGCCAAGGCTTCTGCCATCCACAATCCCATTATCTACGCCATCACTCACCCCGAGCAGAACGTGCAGAAGCGGAAGCGCAGCCTCAAGAAGACCTCCACGATGGCGGCCCCGCCCAAGAGCGAGAACTCAGAGGACGCCAAGTAG

1. CMV (GenBank accession number KR005389)

ATGGACTCTCCTTCAGGACCAAGAGTCTTGTCAAGCTTAACTCAGGATCCCAGCTTCACAACCAGTCCTGCCCTGCAAGGCATTTGGAACGGCACTCAGAACGTCTCCGTAAGAGCCCAGCTTCTCTCTGTTAGCCCCACGACATCTGCACATCAGGCTGCTGCCTGGGTCCCCTTCCCCACAGTCGATGTCCCAGACCATGCTCACTATACCCTAGGCACGGTGATCCTGCTGGTGGGACTCACAGGGATGCTGGGCAATCTGACGGTCATCTACACCTTCTGCAGGAACAGAGGCCTGCGGACACCAGCAAACATGTTCATCATCAACCTCGCAGTCAGCGACTTCCTCATGTCAGTCACTCAGGCCCCGGTCTTCTTTGCCAGCAGCCTCTACAAGAAGTGGCTCTTTGGGGAGACAGGTTGCGAGTTCTATGCCTTCTGCGGGGCTGTCTTTGGCATCACTTCCATGATCACCCTGACAGCCATAGCCATGGACCGCTACCGCATTTTCGAGCAAGGGAAGCGCTCTGTCACGCCGCCACCCTTCATCAGCCCCACCTCGCAGCTCGTCCTGCTAGGCGTCTGGCTTTATGCCCTGGCCTGGAGTCTGCCACCTTTCTTTGGTTGGAGTGCCTACGTGCCCGAGGGGCTGCTGACATCCTGCTCCTGGGACTACATGACCTTCACACCCCAGGTGCGTGCCTACACCATGCTGCTCTTCTGCTTTGTCTTCTTCCTCCCCCTGCTCATCATCATCTTCTGCTACATCTTCATCTTCAGGGCCCGAGGTGTGCCAGAGACCTTCAATGAAGCCAAGGTCGCACTGATTGTCATTCTTCTCTTCGTGCTGTCCTGGGCTCCCTACTCCACTGTGGCTCTGGTGGCCTTTGCTGGATACTCGCACATCCTGACGCCCTACATGAGCTCGGTGCCAGCCGTCATCGCCAAGGCTTCTGCCATCCACAATCCCATTATCTACGCCATCACTCACCCCGAGCAGAACGTGCAGAAGCGGAAGCGCAGCCTCAAGAAGACCTCCACGATGGCGGCCCCGCCCAAGAGCGAGAACTCAGAGGACGCCAAGTAG

1. CMVI (GenBank accession number KR005390)

ATGGACTCTCCTTCAGGACCAAGAGTCTTGTCAAGCTTAACTCAGGATCCCAGCTTCACAACCAGTCCTGCCCTGCAAGGCATTTGGAACGGCACTCAGAACGTCTCCGTAAGAGCCCAGCTTCTCTCTGTTAGCCCCACGACATCTGCACATCAGGCTGCTGCCTGGGTCCCCTTCCCCACAGTCGATGTCCCAGACCATGCTCACTATACCCTAGGCACGGTGATCCTGCTGGTGGGACTCACAGGGATGCTGGGCAATCTGACGGTCATCTACACCTTCTGCAGGAACAGAGGCCTGCGGACACCAGCAAACATGTTCATCATCAACCTCGCAGTCAGCGACTTCCTCATGTCAGTCACTCAGGCCCCGGTCTTCTTTGCCAGCAGCCTCTACAAGAAGTGGCTCTTTGGGGAGACAGGTTGCGAGTTCTATGCCTTCTGCGGGGCTGTCTTTGGCATCACTTCCATGATCACCCTGACAGCCATAGCCATGGACCGCTACCGCATTTTCGAGCAAGGGAAGCGCTCTGTCACGCCGCCACCCTTCATCAGCCCCACCTCGCAGCTCGTCCTGCTAGGCGTCTGGCTTTATGCCCTGGCCTGGAGTCTGCCACCTTTCTTTGGTTGGAGTGCCTACGTGCCCGAGGGGCTGCTGACATCCTGCTCCTGGGACTACATGACCTTCACACCCCAGGTGCGTGCCTACACCATGCTGCTCTTCTGCTTTGTCTTCTTCCTCCCCCTGCTCATCATCATCTTCTGCTACATCTTCATCTTCAGGGCCATCAAGGCCCGAGGTGTGCCAGAGACCTTCAATGAAGCCAAGGTCGCACTGATTGTCATTCTTCTCTTCGTGCTGTCCTGGGCTCCCTACTCCACTGTGGCTCTGGTGGCCTTTGCTGGATACTCGCACATCCTGACGCCCTACATGAGCTCGGTGCCAGCCGTCATCGCCAAGGCTTCTGCCATCCACAATCCCATTATCTACGCCATCACTCACCCCGAGCAGAACGTGCAGAAGCGGAAGCGCAGCCTCAAGAAGACCTCCACGATGGCGGCCCCGCCCAAGAGCGAGAACTCAGAGGACGCCAAGTAG

1. CMVII (GenBank accession number KR005391)

ATGGACTCTCCTTCAGGACCAAGAGTCTTGTCAAGCTTAACTCAGGATCCCAGCTTCACAACCAGTCCTGCCCTGCAAGGCATTTGGAACGGCACTCAGAACGTCTCCGTAAGAGCCCAGCTTCTCTCTGTTAGCCCCACGACATCTGCACATCAGGCTGCTGCCTGGGTCCCCTTCCCCACAGTCGATGTCCCAGACCATGCTCACTATACCCTAGGCACGGTGATCCTGCTGGTGGGACTCACAGGGATGCTGGGCAATCTGACGGTCATCTACACCTTCTGCAGGAACAGAGGCCTGCGGACACCAGCAAACATGTTCATCATCAACCTCGCAGTCAGCGACTTCCTCATGTCAGTCACTCAGGCCCCGGTCTTCTTTGCCAGCAGCCTCTACAAGAAGTGGCTCTTTGGGGAGACAGGTTGCGAGTTCTATGCCTTCTGCGGGGCTGTCTTTGGCATCACTTCCATGATCACCCTGACAAAGACCAACCGCATCTACCGCATTTTCGAGCAAGGGAAGCGCTCTGTCACGCCGCCACCCTTCATCAGCCCCACCTCGCAGCTCGTCCTGCTAGGCGTCTGGCTTTATGCCCTGGCCTGGAGTCTGCCACCTTTCTTTGGTTGGAGTGCCTACGTGCCCGAGGGGCTGCTGACATCCTGCTCCTGGGACTACATGACCTTCACACCCCAGGTGCGTGCCTACACCATGCTGCTCTTCTGCTTTGTCTTCTTCCTCCCCCTGCTCATCATCATCTTCTGCTACATCTTCATCTTCAGGGCCCGAGGTGTGCCAGAGACCTTCAATGAAGCCAAGGTCGCACTGATTGTCATTCTTCTCTTCGTGCTGTCCTGGGCTCCCTACTCCACTGTGGCTCTGGTGGCCTTTGCTGGATACTCGCACATCCTGACGCCCTACATGAGCTCGGTGCCAGCCGTCATCGCCAAGGCTTCTGCCATCCACAATCCCATTATCTACGCCATCACTCACCCCGAGCAGAACGTGCAGAAGCGGAAGCGCAGCCTCAAGAAGACCTCCACGATGGCGGCCCCGCCCAAGAGCGAGAACTCAGAGGACGCCAAGTAG

1. CMIII (delta L) := Opto_mGluR6 (Genbank accession number JB414804.1)

ATGGACTCTCCTTCAGGACCAAGAGTCTTGTCAAGCTTAACTCAGGATCCCAGCTTCACAACCAGTCCTGCCCTGCAAGGCATTTGGAACGGCACTCAGAACGTCTCCGTAAGAGCCCAGCTTCTCTCTGTTAGCCCCACGACATCTGCACATCAGGCTGCTGCCTGGGTCCCCTTCCCCACAGTCGATGTCCCAGACCATGCTCACTATACCCTAGGCACGGTGATCCTGCTGGTGGGACTCACAGGGATGCTGGGCAATCTGACGGTCATCTACACCTTCTGCAGGAACAGAGGCCTGCGGACACCAGCAAACATGTTCATCATCAACCTCGCAGTCAGCGACTTCCTCATGTCAGTCACTCAGGCCCCGGTCTTCTTTGCCAGCAGCCTCTACAAGAAGTGGCTCTTTGGGGAGACAGGTTGCGAGTTCTATGCCTTCTGCGGGGCTGTCTTTGGCATCACTTCCATGATCACCCTGACAGCCATAGCCATGGACCGCATCTACCGCATTTTCGAGCAAGGGAAGCGCTCTGTCACGCCGCCACCCTTCATCAGCCCCACCTCGCAGGTCCTGCTAGGCGTCTGGCTTTATGCCCTGGCCTGGAGTCTGCCACCTTTCTTTGGTTGGAGTGCCTACGTGCCCGAGGGGCTGCTGACATCCTGCTCCTGGGACTACATGACCTTCACACCCCAGGTGCGTGCCTACACCATGCTGCTCTTCTGCTTTGTCTTCTTCCTCCCCCTGCTCATCATCATCTTCTGCTACATCTTCATCTTCAGGGCCCGAGGTGTGCCAGAGACCTTCAATGAAGCCAAGGTCGCACTGATTGTCATTCTTCTCTTCGTGCTGTCCTGGGCTCCCTACTCCACTGTGGCTCTGGTGGCCTTTGCTGGATACTCGCACATCCTGACGCCCTACATGAGCTCGGTGCCAGCCGTCATCGCCAAGGCTTCTGCCATCCACAATCCCATTATCTACGCCATCACTCACCCCGAGCAGAACGTGCAGAAGCGGAAGCGCAGCCTCAAGAAGACCTCCACGATGGCGGCCCCGCCCAAGAGCGAGAACTCAGAGGACGCCAAGTAG

1. CMIII (delta L) – IL1 replaced (Genbank accession number JB414806.1)

ATGGACTCTCCTTCAGGACCAAGAGTCTTGTCAAGCTTAACTCAGGATCCCAGCTTCACAACCAGTCCTGCCCTGCAAGGCATTTGGAACGGCACTCAGAACGTCTCCGTAAGAGCCCAGCTTCTCTCTGTTAGCCCCACGACATCTGCACATCAGGCTGCTGCCTGGGTCCCCTTCCCCACAGTCGATGTCCCAGACCATGCTCACTATACCCTAGGCACGGTGATCCTGCTGGTGGGACTCACAGGGATGCTGGGCAATCTGACGGTCATCTACACCTTCATGCGACACAACGACACTCCCATAGTCCGCGCCTCTGGCCGTGAGCTTTTCATCATCAACCTCGCAGTCAGCGACTTCCTCATGTCAGTCACTCAGGCCCCGGTCTTCTTTGCCAGCAGCCTCTACAAGAAGTGGCTCTTTGGGGAGACAGGTTGCGAGTTCTATGCCTTCTGCGGGGCTGTCTTTGGCATCACTTCCATGATCACCCTGACAGCCATAGCCATGGACCGCATCTACCGCATTTTCGAGCAAGGGAAGCGCTCTGTCACGCCGCCACCCTTCATCAGCCCCACCTCGCAGGTCCTGCTAGGCGTCTGGCTTTATGCCCTGGCCTGGAGTCTGCCACCTTTCTTTGGTTGGAGTGCCTACGTGCCCGAGGGGCTGCTGACATCCTGCTCCTGGGACTACATGACCTTCACACCCCAGGTGCGTGCCTACACCATGCTGCTCTTCTGCTTTGTCTTCTTCCTCCCCCTGCTCATCATCATCTTCTGCTACATCTTCATCTTCAGGGCCCGAGGTGTGCCAGAGACCTTCAATGAAGCCAAGGTCGCACTGATTGTCATTCTTCTCTTCGTGCTGTCCTGGGCTCCCTACTCCACTGTGGCTCTGGTGGCCTTTGCTGGATACTCGCACATCCTGACGCCCTACATGAGCTCGGTGCCAGCCGTCATCGCCAAGGCTTCTGCCATCCACAATCCCATTATCTACGCCATCACTCACCCCGAGCAGAACGTGCAGAAGCGGAAGCGCAGCCTCAAGAAGACCTCCACGATGGCGGCCCCGCCCAAGAGCGAGAACTCAGAGGACGCCAAGTAG

*PRmGluR6, full-length mGluR6 Promoter (10‘154 bp)*

ggtcgaccggtcgaccggtcgaccccctttgcagactccaccaactagaagttagtccactcttcctcatgggccttcgcctctggtccaaagtattaccaaagtcacttaaattaacaagaacagacacacacacacccaagctagaactagcagcactagccagaactcaatttacattttagagaaaaagggggtggaggacagctcctgtagagggaatgatattaacacgttctgggctccgtgcccagcatcgttctgctcctttccaacagtaaaaccttagagcaaaggcacaagtggaaaaaatggactgtggaattcagttaagatactgtccagcaccgaagactgacagaaactaagtttcacctccaggattgaaagcctacaggcgatctgctcaaggccgacttgactagctaacctgaagccggaggcttctttgaccgctgttcgggcagcagaacctggagtcagggcccgaggccctcaccagcagctgaggcctctgcgtgcttccgccaggctctcagccctggcccgcaggttcccggccgttccagctctgccagaaaacccagaaagctcaatgcccagagcgggtaagactaggctcaactgcgcgtgcgcgcgagccacctggtttccactgtggactacatttcccagaaggcactgtgacactcctacccaccctgtatggtgcagagtgggacacaggcgcctaaagactgagaatcaacttttcagttgccaccagctttcaggtttctgtgcaggcttcattcataattacaatggtaatactactaaagaggaaaaagtgagtgtgcattaaaatgttgaagaataaggctctgactgcttagtttcagatagcgaaaggactgtcctctttcattttttaatagaaaattatgctttttctaggctacaaaagatacataacatacacaatttttcattgctggctcatactttgtattaagcaaaaaactgccatattagtcattactgtcatggacaactcagattttcaggggaagcaaacaggtagaaataatttattcattacttaagttggaaatgtctgttttttacaaaaattttttcctgtctttgtccactgtaaaagttctgaagaatgattattcggtctcaacaagatacaaattatgttctctaggtagcaattaacacaaggaacgccttgaggtatgggaggggtgaggaagctcacaagatagaccctggtgcctggaaggaagacagccaactaaaggtcatatcacagtgtcccgggaaccaacttgaagggcttctgctgtacaaatgtgggagaatttcatcgtcagaaggctctgcaaaggtctgaaagtcaccgaactctgtaagattctatcctgcttctattcctgtcaaaatataccagaaggaatggaactaccccctccaaaaaataaataaacaaacaaaccaccaaaccacgcacagacaaagcattcaatacacatgctaaaacataccactttagtttaaggactatagtgattccacactaggtaaggtgctttctgtaggcttttagttaatagttttgtcaagctaaagaagatctccagatggctaaacttttaaatcatgaatgaagtagatattaccaaattgctttttcagcatccatttagataatcatgttttttgcctttaatctgttaatgtagtgaattacagaaatacatttcctaaatcattacatcccccaaatcgttaatctgctaaagtacatctctggctcaaacaagactggttgtgacaggtttgtctctgtcagtttgtgactgttgggctggctcttcctacccctctgcttcttggtttggcctgaacattaattttattttatttttttaattttacctacaatcaatttcacaatgtgtgttgtcattttctcctattgtgtgatattttgtgaacagagaaattcctttgcaacataactgagtatcatgggttagttttttcttcagtagaaggcttcacatgggtcttttctgctctgagtgagagcagctcaatgctgtgagctgacacagcagactgcaatacaacctgttgtgttttataaaaagataaggaggaatgaaatctgtttggtggatgtgtggtcaggtgtggggaaagggggtgcctccacgggcccatgctgaggctttccttccccgtgaaggaccagcctcaggacagtatgttatagaatagagtttattcagggcatgaggaggggagttgagagaaaggcagagagagagagagagagagagagagagagagagagagaatatatagaggagtagaggctgaccatgagcacagggagagagggggagaggggaatggggagggagaataaggaacaggagcaagagagcaagaacaagagagacaagagaaggcaagctgcccctgttatagtgagtcaggcatacctggctattgccaggtaactgtggggcggatcccagactaaatgccaacacaaccagaggaaggggagatgtgtttggtgttccttcgtctccctcagcacactgtgtgtgcctgttctctgaaaaatgctctggccatttctttttaactcctccgtgctgaactggaacccagttgtgcaagcgaggcaggcagtctaccgtagcgctagatttttacttttaaaccgggatctcgctttgcattaatgccctgcttccacatctgcttacagcttagtgtgttgttttgcttttatccccctcacactctcagtttttcctgtggagtttcacacacaaattttcagcagggacaccctttctggttccttgatattactgctgttgtcattttgacattgttcttcgtctgggctccagctactgttctttctacctcccagacaccaacattgttcttcactcaggtttctgcccatgcatcatctaccttgctgtgtattcaactggatatccatatgcaaatggttgaatttggacccaacatcatattacactcaaaaattccctcaacatggatcaacgatctaaatgttagcgctagaatcacaaaacactaaaaataaaacacgggagtgtttagtgatgtcttagttatggtttctattgctgtgataaaacactgtgattaaaagcagcagcagtggggtgaggcagggtaggcaggaaaggttcaatctcagcttagaactctctctctctggtcatgctccatcagtgaatggagtaagagcaggaacttgaggcaggagctgatgcagaggcccagaaggaaggaacctgcttactggcttgctccttgtggcttgctcaacctactttcttgttgactccaggaccacctgccaagggctggcacctcccctaggggactggaccctcccacttcaatcattaatcaagaaaatgccccacagggggcattttcaattgtgactctctcttctcagaggactcttgtttgttaacaaaaaactaaccagggcaggtataaatcttcatgactttggaattgcctgtgggatctcagatgtgctatccaaacacaaacaataaaagaaaaatgcaatttgaatcttaacaaatgttttgaatcttaaaatgttatgtattatgaagaaagtaaaccgataactcacagacaggaacaaaaatctttgcaagtcaaaagtttaataagtccaggctttacaccttaacaagaagactgagtctgtggctacataccgtggcacatattactactagagcatgggatgcccctggtaacggcaacttctggggaccacgtggatgtccggggactgtgcataacttgtcccacccctcactggatgcggcactctagagagctggccccatctctcacctatggcggcactctggagagtgggccgggcagcacagtggagctgctcctggcttcgagggtagagatgagccagctccaaggatgtgagtgtgggagagctgaccctgccacttgtctgccatgggtagcacaggtgcagatgtgatacacacacacacacacacacacacacacacacacacacacactgccccgcactactcctgaagtcaggagctagtcccacccctaccagctacagctctcagaacagtcccgggaccttgtctggagagcacagcagaactaaccctggtgttgagggtgcaggaaacccagccccaagagtgaaagctcggaaaagctggctccatcattcatctgctgtgaggtgccatgggtgtgcaggtgatgctctccccacctcttcactccctgccacctaaggcagtagggacagctggtccccagggacatcagagtgggagagctggctctgctcctcactggctgtagaactcagaatgggccctgcatcttgtctgggcagcacaatagggctggccttgttggagggagagaggatggggcagcccagagggtagagtgtaggagaggtggccccgacacttgtctggtgtgaggtggtgtggatgtaggggcaatgccctccctgggcccctcactgcctgaggcagtcaggagaactgaccccagggtcatgagagcaggtgagctggccctgctcccactggctgcacttcagagagcaggccctgaatctcctctgggcagcatagtcgcactagcactggtagagggggcacgggtgaactccaccccccatcccccaccaggttaagagcatgggagagctggccttgccacttgtcttgtgtgaggtgggtgcggggattatgtcctcttcccccacctgcagtggctgggagagttgaccctggaccatgagagggagagagagctaatggcccctcattggctatagcacttgggtgagtgggcctgcacctcacctgggcaatacagtggagctggccctgatggtgaaagtatgggtgagttagtccagagggtatgagagtgtgagaaatggcccagcctctcacaggctgcagcacctgggagagcgcaccctgaaccttgaatggatagctaggtggagcggctctggaggaatgggtgcaaatgacccatcctgagggcaagagagcaggagtgctgaccttgcctcctgccaatggagggaggtattgactggcctagctggaagagtgctggagagtttactctagtggtgtgggtaagggagagctggcaagctgaccagctcagctactacccagggcaagatcctgggctctgagccagcccaccccaaaatcgatatcatctgtgaacagttgacatgcatgaaaggggcatccttgctattctaaaactgcaggctctccatgacacagagcaacaacaggataacccagaggggtctcaatgaagatccaatattgatggtatcacagaagctaaagacttcaaaccagaccgttgactcattataatgaacaccttaccttcaagtgaagatgtgtggacagagggaaatactgtaggacacactgtgacacactacagcttgcatggtgagatgttttctatgctttgttttgttgttgttgttggtggtggtggtggtggtggtggtggtgtggtggtggtagtggggtgtgtgtgtgtgtgtgtgtgtgtgtgtgtgtgtattattttggggggaggttgcgagggtgaagaatagatatgaggagatggggagatgagcagaactggggtgcatgatgtgaaactcacaaagaatcaataaagtttttaaaaactcagaaactaagccgggcggtagtggcgcacgcctttaatcccagcacttgggaggcagaggcaggtggatttctgagttcaaggccagcctggtctacaaagtgagttccaggacagccagggatacacagagaaaccttgtcttaaaaaacaaacaaacaaacaaacaaacaagaaacaaaaacaaataaaacaaaaaaccctcagaaactagttttaaagcttatcaaagcagactctactcgctgttttactgaatttcatcaagctaagtactttaggggagagagaatctcctcctcagcctgcagtttctatactactggactgtaaaattcccgagagtaagatatgaatcctgggcctgtaaattatatttaaactaatatatattcaaaacagtgaattatagggaaaaaaagaaaactccgtttatatggtgcttcattcacccttagtgagctatttccctggttgccaccaggccaccctgtggtggcagcactgagtactcctagctgccaagtcagtctttgcacagcacattcacatggcgatcgaaccaaagagcgtgtttaatggtgcagagctatattgaaggaagcttgcatagctgggtgtcaacaagtgctgatggctgattgttttaataccccatcctgctacattgaaaggtctgcagttgccttgggcttggcagaggagcctagcggaaagacaggctgtcaaagcagcagtgggatgagggatgaggtgatagttagtcctccctgtcaacttactagtttcgaatcacctgggagacacattgctgagtgtaactgtgaggggctctctagggaggttaaactgagggggaaacacccaccctgaatgtgcgtggcactataactgaatgcaaacgggaaagaaagaaacaagctggctggggagcaccagaattcatctctcctttcttcctgactgtggacaccatgtgaccagctgcctcacactctttccagcaaaccttgctgtcatggaagactgtgttccctccaactgtgagccagaatagtccttctcttgtatcacttgtctttgtcaggtattttgtcatagcaatgagaaacataacccagtatggagttacttagttacacttgctccatactggtccacgcaggaccctaaggcctctgtggacattctcagttgcagacatcatgcttttcaacaccttgtgctagagatggtgaagaatgctccaactctcctgcctacatgttctctaaaagtgagaaggtggacagcactcttagcactcctagtcagagggcagaggtttgacccatacattgaaccctcaaaggtatagtcttaagtctatttgtgtgcacatgcttgcacacacacacacacacacacacacacacacacacacacacacacacgtgcacgctcccacacagaagctctgcttggatagtctcctgcagtgtcacccactctggtcaagccccactaagctggcttccatcacaggaatgaactgctctgggtgtgacaagagaaatcggaggatagtggttatatttctgctgcttgctctctccaccagtcatgtccagatctctgctgccaccctaatccaccctgactaatgcactgaagagcccattaatccctggaggctggggctcagcaactgtctccaagatgcctttgctgtccagcatcagagagctcaatcctgtcctctgttgacaatgatgggaaaatatctttgggttgaacatcttcacggtgtaaatcagttccagagagctaggaaactcagaaatgatgtggggagacaactgagggctcctgacccacatgggagcttcagggtgaactaaccccatcttcccccctccaagccagtgggtaggctggtgtttcacaccactctgaaatgcaaatctagttgctgacaaaggccagctgcagagccttagggccatagggcagccagtcatttcctgaggtgtctatttgtctgtctgcagatggagagattctctgcaaggctttggtgtgtttgctgctgctgaaggtctgttcagcattgtttccagccttaccaaggcttcttgcatctgtccttcagattcactgtgctggcacaccctggctggctcagctcctatcatctgccacttacgggtttgcttcagagaaagttggggtggcttttatgcagctgcattaaaaagaactactaaactctgataagatggctcagctggtaaaagtgcctgctgccaagactcacaacctgtgttcagtcctcaggaccaacatggtgaaaggtgataggttatttctctgccgctagtgaaatgagccaagttgggatatgttaaaggcaggtttattgggaagctgctcttaggtgagttcacagacccggaggattgagggcagggcagttgccatggggggaagaggggaggtgagggagaatagaaagcgagaaagggggcacagatgtcccgacccgcaggacccagttattcagggggtctgagggagaccttgcctgaaagagaaacgggcgggaaataagagacagaccaagtagatccatcaaggtctgtttattgagagtaaggttacagaatataagcggcaaggaggaaggaagtaaagagggagaaacttgcccgtgcctcagcccgcaggcaggggtggttctgcacaactgcccgggaaggtgctatctactcttagctcaggggacattctgtgttttttcacagaaagtttgcagatactattatctgcccttgatgttgtgtcagctgtcatttcaaaaggtcggaagtctctcctccaggagggagcggaactttggcttatgactcagtgtcagtccccaacatctctcaaaaggtccgaagtttctcacgaaggaaggggagctttggcttatggctcaatgccggtctccaacacagagagagaagagaaggaacagaaggaagagagaaagagaccaaaatgtctggatcacatagggaagaacctctgggaaaaaggcagcccagcccctgggctggaaagttcagggtgggaggcagggtatgtcaggtagggactgggggatgctgggagatccctgaagtcaggtctgctttgatatgcaaactatgcaccttgtcccggtcccaaaccaaaagggagagaactaactctggcgtgagagggcatgtgccgcatatcacacacacacacacacacacacacacacacacacacacacacacacacacacacaaaaccatgcacgctcgcacacgatagataatacatacaccaatatctgaaaagagaaaaggttctagtggtcaggacagagaatgaaaacggcaggaaggcaagaaagtttgagaacgtagggggtggggtagggagacactacgagtggaataagccacgtttggagaacgtctaggcagatacagaaatgcagaacacagagagaccgagaccagagcagcgtcagaccggctgcaaggctcttgttaggggctttagaaacacctgtgtgctctcccggaagcctggtgcagtcagagaggaagcttgcttcccagacagagatgacacagtttcacaacctgtcagaccaccttgcaggagagactgaaccccagcaaccagaaccacttggctatgcatgtccttttctgtttaaacctaagtctctgaagaccgaccaggggagtccctggacttctttgttcctcttctcggggtggcgggactgattgtgtaaatctcttatctccaactttcactcttatctgtctctttaatcggcatattgaggatgagtggccaagcttattggtgttgctgggtcagacaatttaaaggcagtctaggggagaagcagacccagggagtcagagaggcagagagagaagagagcccttcctccactctcaagctctggagggggtctctgccctcaccctcatccctccccagaatccttaaatcctctagactgtagctctgattttacagctgtcacagactcgtcctactagccagaggttggctcaggtaagcaccactggggaggtagcctagggtgcgctggggtgggtccagaggaagagctgcccagaactgtgggggaaggagcgggaccgaccatcaacagggggacttttcagggagaatgagagcaatcctctggaggcctgggagaggctgctgagttgctggtgcgcgagtcaccaacttttcctgcgctctcggtgtccggccagaatcccgaagtggcagctgagcacggggtggcagcttcgtccgcc

*mGluR6/sv40 promoter enhancer sequence (419 bp)*

gatctccagatggctaaacttttaaatcatgaatgaagtagatattaccaaattgctttttcagcatccatttagataatcatgttttttgcctttaatctgttaatgtagtgaattacagaaatacatttcctaaatcattacatcccccaaatcgttaatctgctaaagtacatctctggctcaaacaagactggttgctcgagatctgcgatctgcatctcaattagtcagcaaccatagtcccgcccctaactccgcccatcccgcccctaactccgcccagttccgcccattctccgccccatcgctgactaattttttttatttatgcagaggccgaggccgcctcggcctctgagctattccagaagtagtgaggaggcttttttggaggcctaggcttttgcaaaaagctt
