# Supplementary figures and images for: Restoring the ON Switch in Blind Retinas: Opto-mGluR6, a Next-Generation, Cell-Tailored Optogenetic Tool
Source: PLoS Biol. 2015 May 7;13(5):e1002143. doi: 10.1371/journal.pbio.1002143 (PMC4423780; doi:10.1371/journal.pbio.1002143)

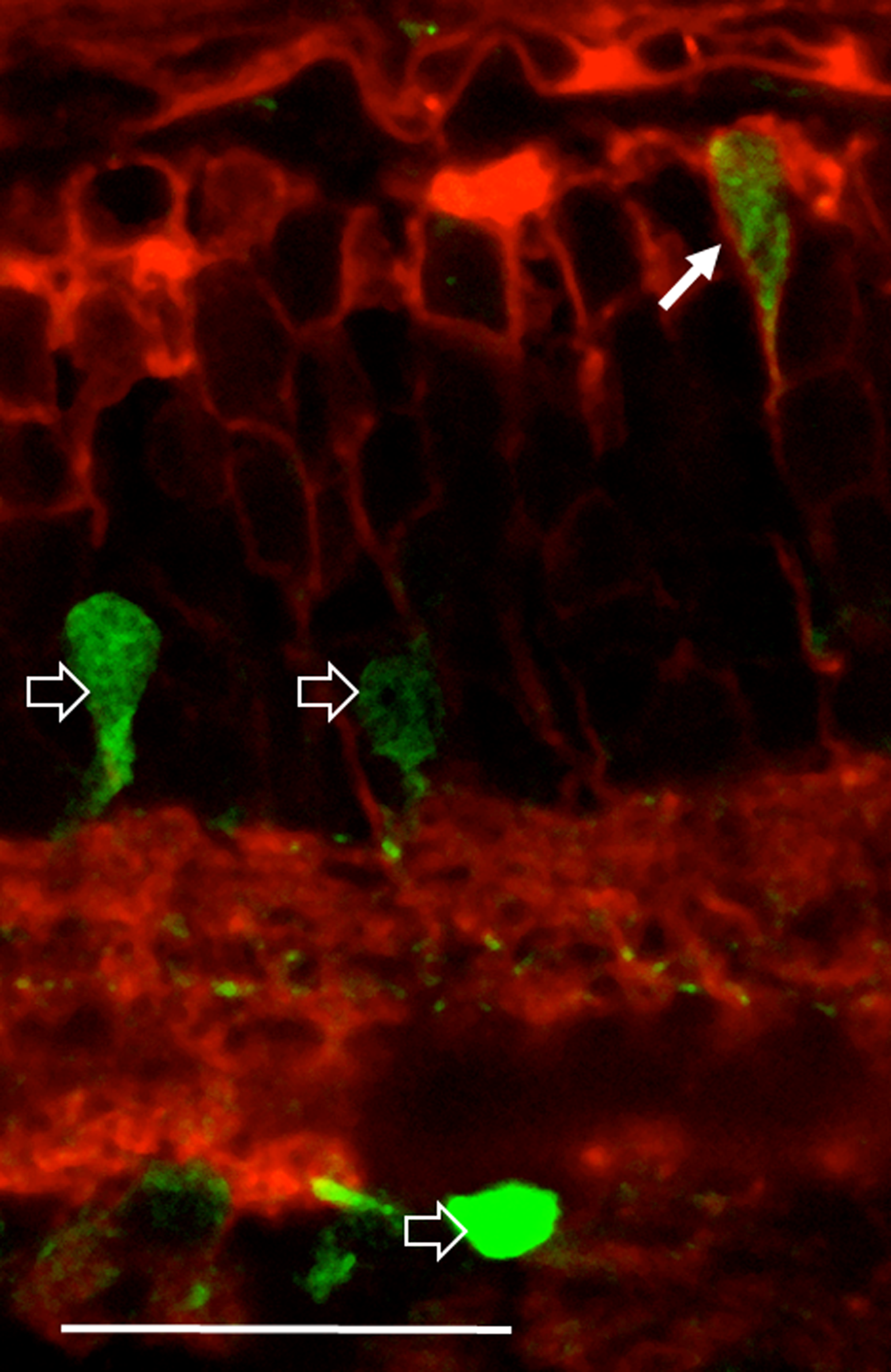

Supplement: S1 Fig — Immunolabeling against Gαo (red) and the Opto-mGluR6 reporter gene TurboFP635 (green) of an rAAV-GRM6/sv40-Opto-mGluR6-TurboFP635 transduced rd1 retina clearly indicates, in addition to ON-bipolar cell labeling (solid arrow), nonspecific labeling of amacrine and RGCs (open arrows). Scale bar, 50 μm. (TIF) [file pbio.1002143.s003.tif]

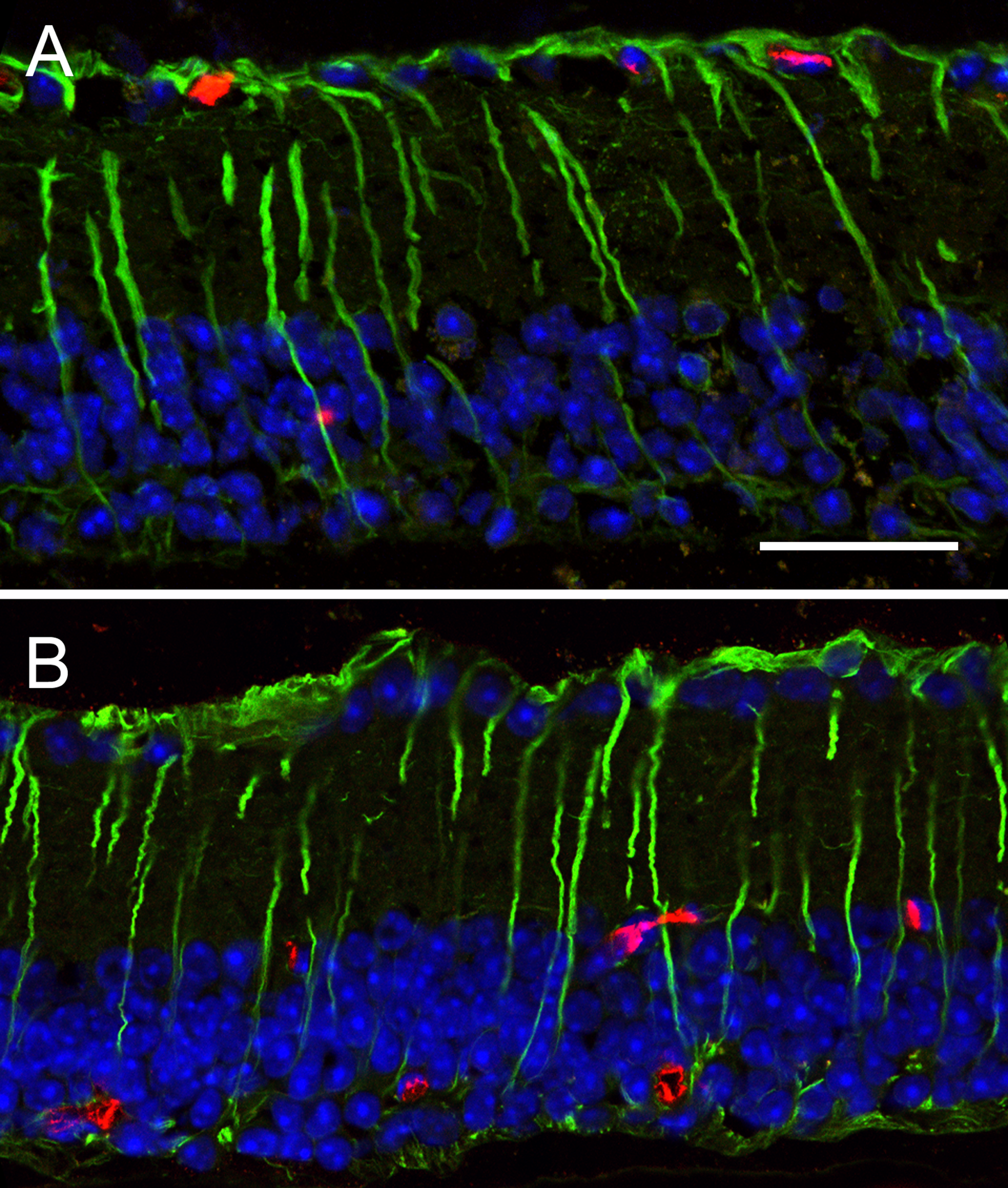

Supplement: S2 Fig — CD45 (red), a general marker for immune cells, and GFAP (green), an astrocyte marker, were used to label retinas of FVB/N mice (A) intravitreally injected with pAAV2.2(Y252,272,444,500,704,730F)-GRM6/sv40_Opto-mGluR6_IRES_ TurboFP635 and (B) rd1 littermates 8 mo after virus injection. As exemplified in the above confocal micrographs, we observed no CD45 expression in injected and noninjected eyes indicating limited presence of immune cells (red areas indicate nonspecific blood vessel labeling of secondary antimouse antibody). We also observed no change in the number of Müller glia cells (green), known to increase upon retinal injury. Nuclei are labeled by DAPI (blue). Scale bars, 20 μm. (TIF) [file pbio.1002143.s004.tif]

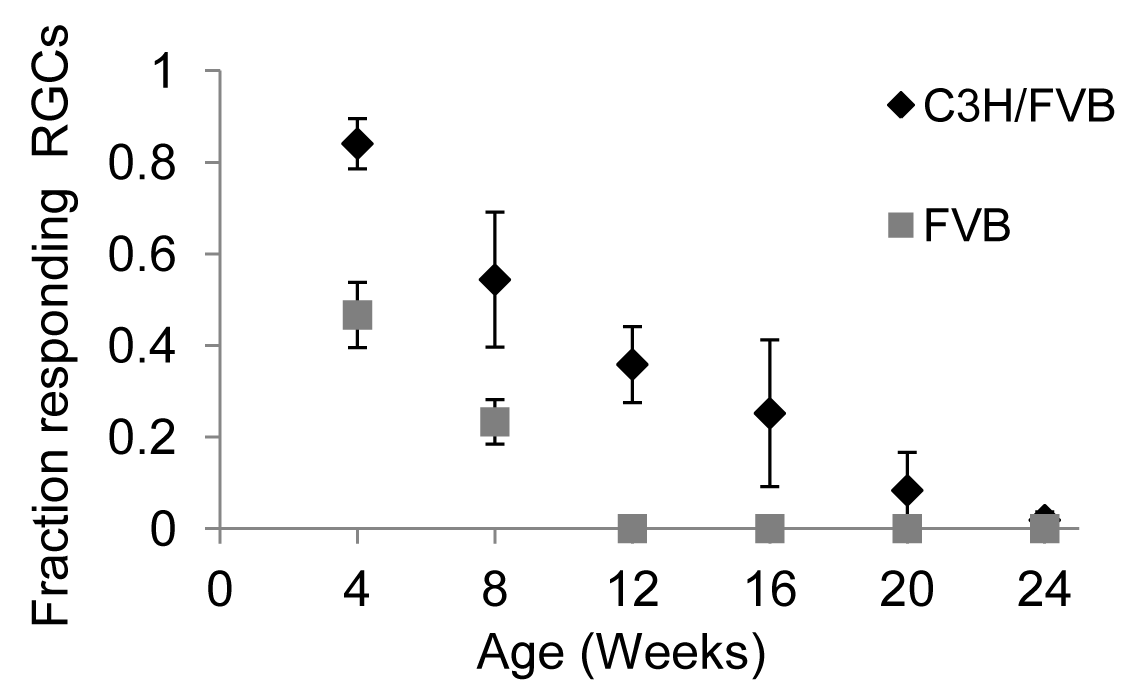

Supplement: S3 Fig — During cell attached recordings, a 1-s light step stimulus (5.4 x 10^16^ photons s-1 cm-2) was projected over the receptive field of the RGC. The stimulus was presented five times to evaluate if action potentials were modulated in a consistent manner. To be classed as light sensitive, spike-time histograms (100-ms bins) generated from all five traces had to have a significant change in firing frequency between at least two bins generated before and after the onset of the light stimulus. In both strains, the fraction of RGCs responding to the light stimulus decreased sharply with age, with a faster attenuation in FVB mice, which had no RGC responses by 12 wk of age compared to C3H/FVB mice, which lost RGC light responses by 24 wk. (TIF) [file pbio.1002143.s005.tif]

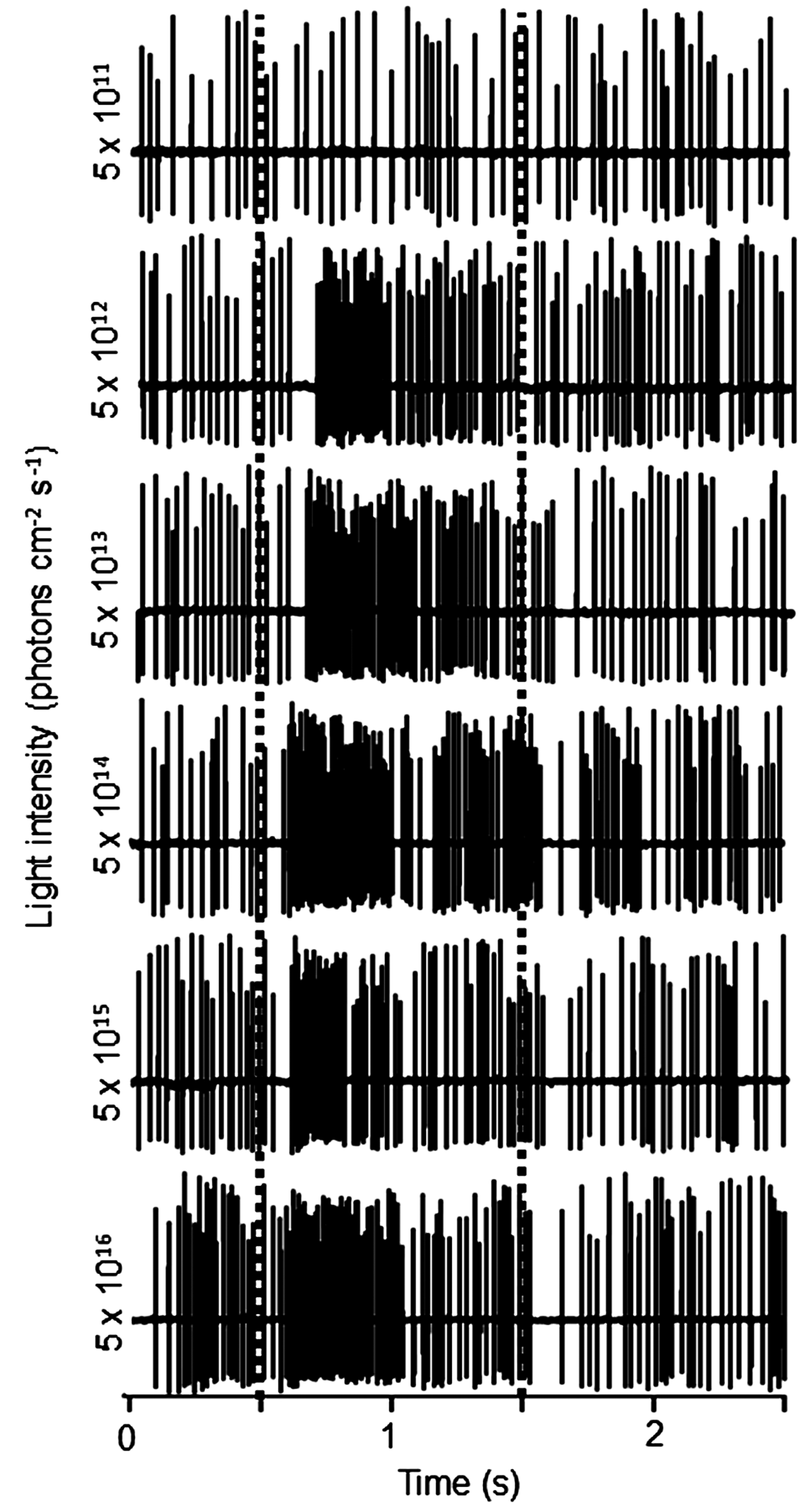

Supplement: S4 Fig — Extracellular recordings from an ON-RGC in a transgenic rd1_Opto-mGluR6 retina. Light flashes of various intensities were presented for 1 sec from 0.5–1.5 sec (between the broken vertical lines). The cell starts responding at a light intensity of 5 x 10^11^ photons cm-2 s-1. As the light intensity increases, the response strengthens. To generate the light intensity response curve depicted in Fig 4C, the spikes for each RGC were counted over the period of the light stimulus using a threshold detection routine written in IgorPro software. For cells with baseline activity, as in the given example, a corresponding trace with no light flash was recorded and the given spike count subtracted from all counts recorded during light stimulation. The spike count was subsequently scaled to a maximum of 1 and plotted against a logarithmic light intensity scale. Average maximum spike counts per light pulse: transgenic retina, 49 ± 18, rAAV-transduced retina, 15 ± 3 spikes (mean ± SEM). Note that the response latency increases with decreasing light intensity. (TIF) [file pbio.1002143.s006.tif]

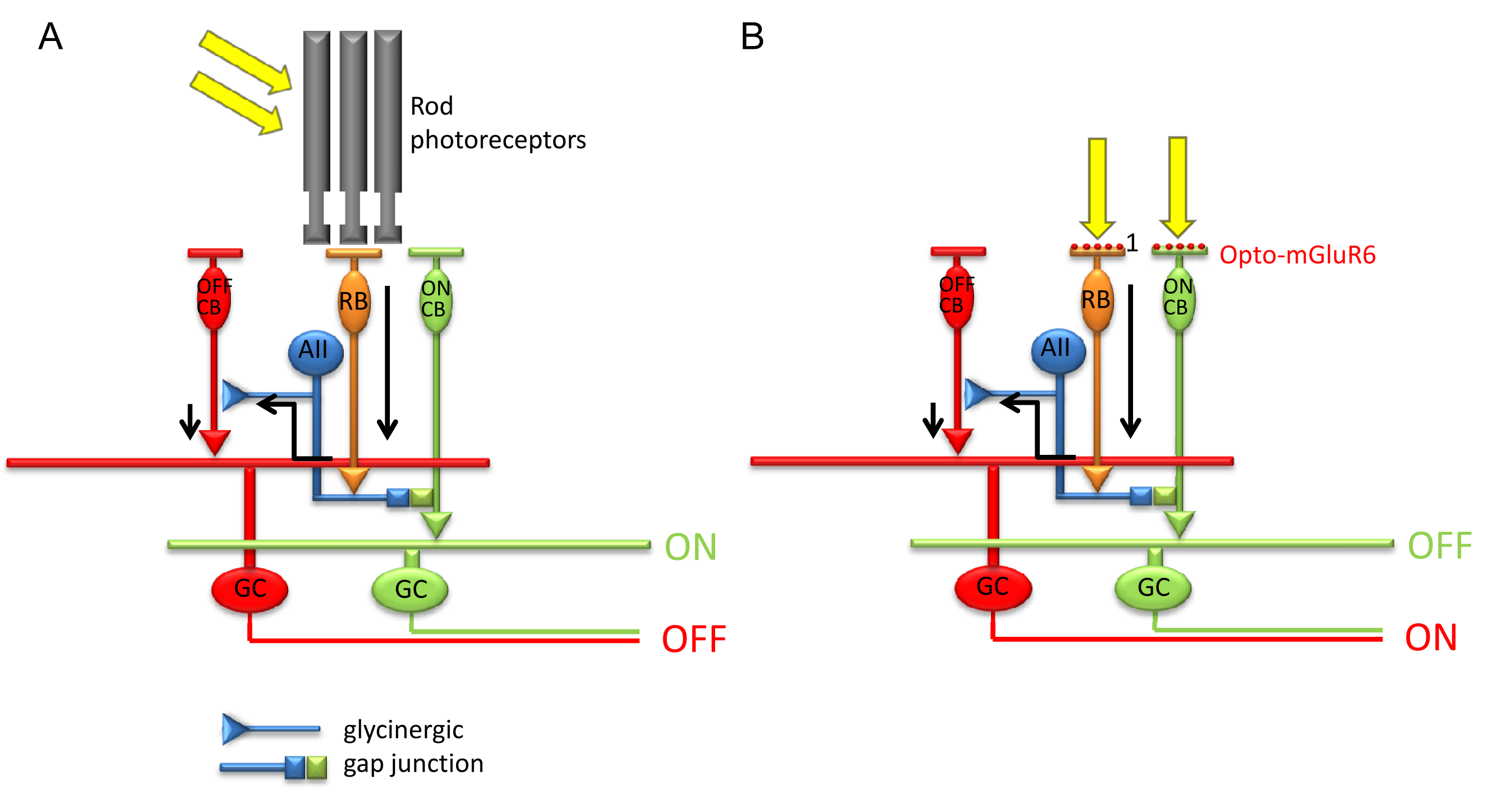

Supplement: S5 Fig — There are two classes of bipolar cells in the retina, ON bipolars and OFF bipolars, which provide parallel information channels responding to increases versus decreases in light intensity. Whilst cone bipolar cells (receiving input from cones) are divided into ON and OFF type (ON CB and OFF CB), rod bipolar cells (RB), receiving input from rods, are all of the ON type. Since rod bipolar cells are by far the most numerous ON-bipolar cells, the majority of Opto-mGluR6 expressing cells will be rod bipolar cells [81]. (A) illustrates the intact rod pathway: upon illumination, the rod bipolar cell (RB, orange) is depolarized, which subsequently depolarizes the AII amacrine cell (blue). In turn the cone ON-bipolar cell (ON CB, green) is also depolarized through a sign-conserving electrical synapse, while the cone OFF-bipolar cell (OFF CB, red) is hyperpolarized through a sign-inverting glycinergic synapse. Finally, ON ganglion cells (GC, green) are depolarized and OFF ganglion cells (GC, red) are hyperpolarized by light. (B) illustrates the degenerated pathway recovered through Opto-mGluR6 (red dots) expressed in ON-bipolar cells: upon illumination, rod bipolar cells and subsequently AII amacrine cells are hyperpolarized. The cone ON-bipolar cells are also hyperpolarized, directly by light and indirectly by the AII amacrine cells, and the cone OFF-bipolar cells are depolarized through the sign-inverting gap junction from AII cells. As a consequence, native ON-RGCs (green) are now hyperpolarized (now OFF-RGCs), and native OFF-RGCs (red) are now depolarized (now ON-RGCs). Black arrows indicate how rod bipolar cells feed into the native OFF circuitry. (TIF) [file pbio.1002143.s007.tif]

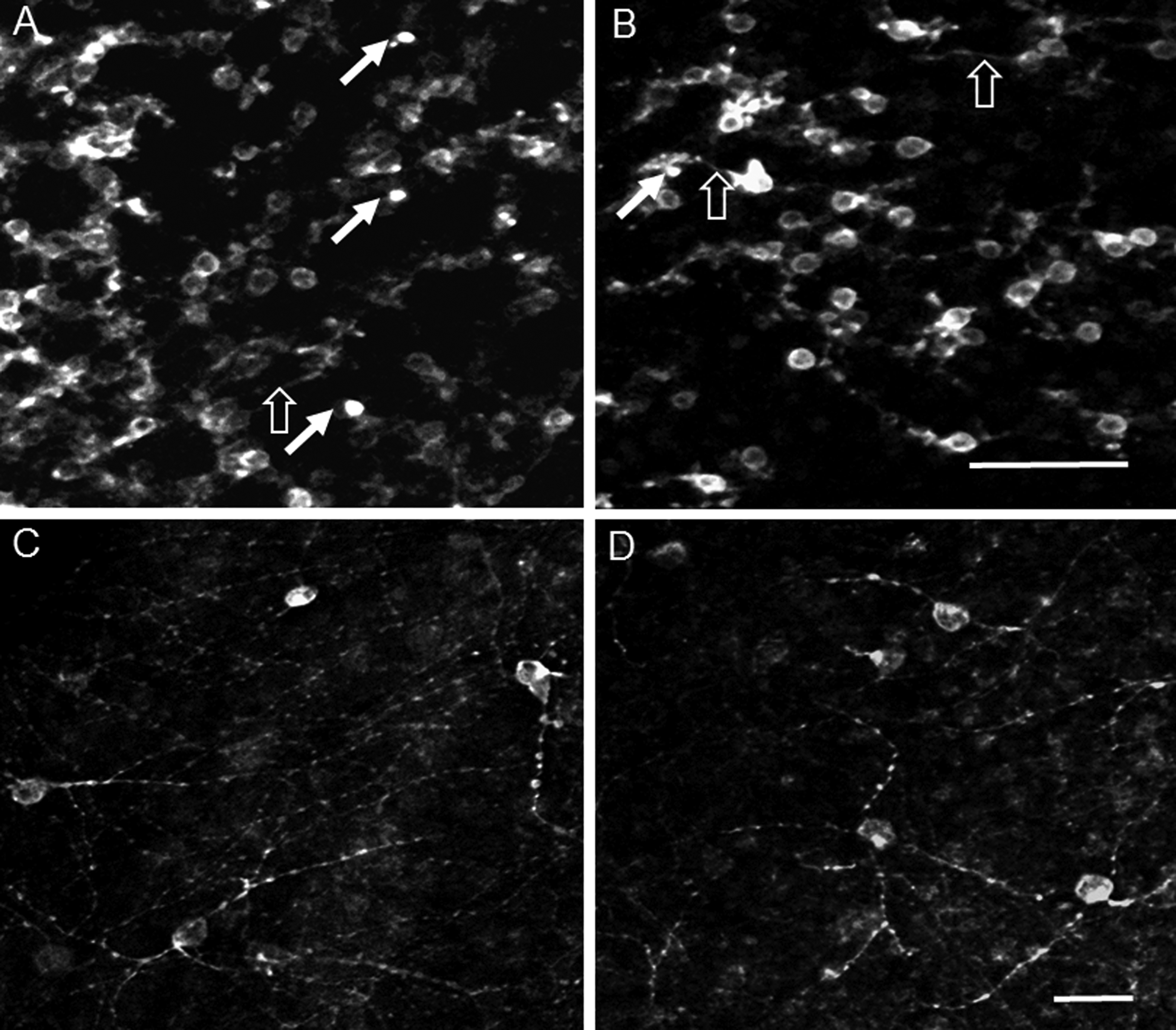

Supplement: S6 Fig — (A,B) Micrographs were taken from the ventral periphery of six OPN1SW-labeled rd1 retinas, three at p180 and three at p280, and the density of labeled photoreceptors counted. Counts were 329 ± 89 cells/mm2 for p180 retinas (example shown in A) and 231 ± 41 cells/mm2 for p280 retinas (example shown in B). Surviving cones expressed opsin in their somas and typically grew long dendritic processes (empty arrows). They had no cilia, although sparse round opsin-labeled structures may represent remnant outer segments (solid arrows). (C,D) Rd1 retinas at p180 (C) and p280 (D) were labeled for opn4. Micrographs were taken in the midperiphery of three retinas from each age group and the number of melanopsin-labeled cell bodies counted. Counts were 22 ± 5 for p180 retinas and 19 ± 3 for p280 retinas. Scale bar is 50 μm. (TIF) [file pbio.1002143.s008.tif]
