# Supplementary material for: Restoring the ON Switch in Blind Retinas: Opto-mGluR6, a Next-Generation, Cell-Tailored Optogenetic Tool
Source: PLoS Biol. 2015 May 7;13(5):e1002143. doi: 10.1371/journal.pbio.1002143 (PMC4423780; doi:10.1371/journal.pbio.1002143)
Supplement: S1 Text — (B) Amino acid sequences of all constructed and functionally tested chimeras. (DOCX) [file pbio.1002143.s010.docx]

**S1 Text**

1. **Overview of amino acid (AA) sequences of the different melanopsin-mGluR6 variants and terminology**

Underlined AA residues are from the intracellular loops of mGluR6, other AA residues are from melanopsin. Four versions of IL 2 replacement at junction (c) have been tested (see Figure 1A for terminology of junction sites). The shaded AA residues represent the most conserved AA sequence motif between TM3 and IL2 in the GPCR family, the DRY site. Notably, additional functional variants of the DRY site include DRIY or NRIY. CM is an abbreviation for chimera.

Type I: TLTAIAMKTNRIYRIFE (CM I and CM II)

Type II: TLTAIAMDRIYRIFE (CM III and CM IV)

Type III: TLTAIAMDRYRIFE (CM V and CM VI)

Type IV: TLTKTNRIYRIFE (CM VII)

Two versions of IL 3 replacement at junction (e) have been tested:

Type 1: YIFIFRARGVPETF (uneven numbers)

Type 2: YIFIFRAIKARGVPETF (even numbers)

All of these variants yielded functional chimeras with no significant differences in their ability to activate GIRK conductances. However, chimeras with a Type 1 IL3 replacement always yielded higher GIRK activity. Therefore CM VIII was not constructed. Also, Type I and Type IV IL2 replacements yielded clearly smaller currents than Type II and Type III IL2 replacements.

One version of IL1 replacement has been tested in CMIII (junctions (a) and (b)):

TVIYTFMRHNDTPIVRASGRELFIINLA

Splice sites of junctions (d) and (f) were always the same (Figure 1) except for subsequent deletion of a leucin (L) residue at junction d in CM III (ΔL). This was done after realizing a better primary sequence alignment when using *Xenopus laevis* melanopsin PPPFISPTSQ(L)VLLGVWL).

1. **Amino acid sequences of all constructed and functionally tested chimeras**

The marked AA correspond to IL1, IL2, IL3 and the C-terminus . The DRY site and its functional variants are marked in bold.

**CM I** MDSPSGPRVLSSLTQDPSFTTSPALQGIWNGTQNVSVRAQLLSVSPTTSAHQAAAWVPFPTVDVPDHAHYTLGTVILLVGLTGMLGNLTVIYTFCRNRGLRTPANMFIINLAVSDFLMSVTQAPVFFASSLYKKWLFGETGCEFYAFCGAVFGITSMITLTAIAMKT**NRIY**RIFEQGKRSVTPPPFISPTSQLVLLGVWLYALAWSLPPFFGWSAYVPEGLLTSCSWDYMTFTPQVRAYTMLLFCFVFFLPLLIIIFCYIFIFRARGVPETFNEAKVALIVILLFVLSWAPYSTVALVAFAGYSHILTPYMSSVPAVIAKASAIHNPIIYAITHPEQNVQKRKRSLKKTSTMAAPPKSENSEDAK

**CM II** MDSPSGPRVLSSLTQDPSFTTSPALQGIWNGTQNVSVRAQLLSVSPTTSAHQAAAWVPFPTVDVPDHAHYTLGTVILLVGLTGMLGNLTVIYTFCRNRGLRTPANMFIINLAVSDFLMSVTQAPVFFASSLYKKWLFGETGCEFYAFCGAVFGITSMITLTAIAMKT**NRIY**RIFEQGKRSVTPPPFISPTSQLVLLGVWLYALAWSLPPFFGWSAYVPEGLLTSCSWDYMTFTPQVRAYTMLLFCFVFFLPLLIIIFCYIFIFRAIKARGVPETFNEAKVALIVILLFVLSWAPYSTVALVAFAGYSHILTPYMSSVPAVIAKASAIHNPIIYAITHPEQNVQKRKRSLKKTSTMAAPPKSENSEDAK

**CM III** MDSPSGPRVLSSLTQDPSFTTSPALQGIWNGTQNVSVRAQLLSVSPTTSAHQAAAWVPFPTVDVPDHAHYTLGTVILLVGLTGMLGNLTVIYTFCRNRGLRTPANMFIINLAVSDFLMSVTQAPVFFASSLYKKWLFGETGCEFYAFCGAVFGITSMITLTAIAM**DRIY**RIFEQGKRSVTPPPFISPTSQLVLLGVWLYALAWSLPPFFGWSAYVPEGLLTSCSWDYMTFTPQVRAYTMLLFCFVFFLPLLIIIFCYIFIFRARGVPETFNEAKVALIVILLFVLSWAPYSTVALVAFAGYSHILTPYMSSVPAVIAKASAIHNPIIYAITHPEQNVQKRKRSLKKTSTMAAPPKSENSEDAK

**CM IV** MDSPSGPRVLSSLTQDPSFTTSPALQGIWNGTQNVSVRAQLLSVSPTTSAHQAAAWVPFPTVDVPDHAHYTLGTVILLVGLTGMLGNLTVIYTFCRNRGLRTPANMFIINLAVSDFLMSVTQAPVFFASSLYKKWLFGETGCEFYAFCGAVFGITSMITLTAIAM**DRIY**RIFEQGKRSVTPPPFISPTSQLVLLGVWLYALAWSLPPFFGWSAYVPEGLLTSCSWDYMTFTPQVRAYTMLLFCFVFFLPLLIIIFCYIFIFRAIKARGVPETFNEAKVALIVILLFVLSWAPYSTVALVAFAGYSHILTPYMSSVPAVIAKASAIHNPIIYAITHPEQNVQKRKRSLKKTSTMAAPPKSENSEDAK

**CM V**

MDSPSGPRVLSSLTQDPSFTTSPALQGIWNGTQNVSVRAQLLSVSPTTSAHQAAAWVPFPTVDVPDHAHYTLGTVILLVGLTGMLGNLTVIYTFCRNRGLRTPANMFIINLAVSDFLMSVTQAPVFFASSLYKKWLFGETGCEFYAFCGAVFGITSMITLTAIAM**DRY**RIFEQGKRSVTPPPFISPTSQLVLLGVWLYALAWSLPPFFGWSAYVPEGLLTSCSWDYMTFTPQVRAYTMLLFCFVFFLPLLIIIFCYIFIFRARGVPETFNEAKVALIVILLFVLSWAPYSTVALVAFAGYSHILTPYMSSVPAVIAKASAIHNPIIYAITHPEQNVQKRKRSLKKTSTMAAPPKSENSEDAK

**CM VI**

MDSPSGPRVLSSLTQDPSFTTSPALQGIWNGTQNVSVRAQLLSVSPTTSAHQAAAWVPFPTVDVPDHAHYTLGTVILLVGLTGMLGNLTVIYTFCRNRGLRTPANMFIINLAVSDFLMSVTQAPVFFASSLYKKWLFGETGCEFYAFCGAVFGITSMITLTAIAM**DRY**RIFEQGKRSVTPPPFISPTSQLVLLGVWLYALAWSLPPFFGWSAYVPEGLLTSCSWDYMTFTPQVRAYTMLLFCFVFFLPLLIIIFCYIFIFRAIKARGVPETFNEAKVALIVILLFVLSWAPYSTVALVAFAGYSHILTPYMSSVPAVIAKASAIHNPIIYAITHPEQNVQKRKRSLKKTSTMAAPPKSENSEDAK

**CM VII**

MDSPSGPRVLSSLTQDPSFTTSPALQGIWNGTQNVSVRAQLLSVSPTTSAHQAAAWVPFPTVDVPDHAHYTLGTVILLVGLTGMLGNLTVIYTFCRNRGLRTPANMFIINLAVSDFLMSVTQAPVFFASSLYKKWLFGETGCEFYAFCGAVFGITSMITLTKT**NRIY**RIFEQGKRSVTPPPFISPTSQLVLLGVWLYALAWSLPPFFGWSAYVPEGLLTSCSWDYMTFTPQVRAYTMLLFCFVFFLPLLIIIFCYIFIFRARGVPETFNEAKVALIVILLFVLSWAPYSTVALVAFAGYSHILTPYMSSVPAVIAKASAIHNPIIYAITHPEQNVQKRKRSLKKTSTMAAPPKSENSEDAK

**CM III ∆L** MDSPSGPRVLSSLTQDPSFTTSPALQGIWNGTQNVSVRAQLLSVSPTTSAHQAAAWVPFPTVDVPDHAHYTLGTVILLVGLTGMLGNLTVIYTFCRNRGLRTPANMFIINLAVSDFLMSVTQAPVFFASSLYKKWLFGETGCEFYAFCGAVFGITSMITLTAIAM**DRIY**RIFEQGKRSVTPPPFISPTSQVLLGVWLYALAWSLPPFFGWSAYVPEGLLTSCSWDYMTFTPQVRAYTMLLFCFVFFLPLLIIIFCYIFIFRARGVPETFNEAKVALIVILLFVLSWAPYSTVALVAFAGYSHILTPYMSSVPAVIAKASAIHNPIIYAITHPEQNVQKRKRSLKKTSTMAAPPKSENSEDAK

**CMIII ∆L with IL1 replaced** MDSPSGPRVLSSLTQDPSFTTSPALQGIWNGTQNVSVRAQLLSVSPTTSAHQAAAWVPFPTVDVPDHAHYTLGTVILLVGLTGMLGNLTVIYTFMRHNDTPIVRASGRELFIINLAVSDFLMSVTQAPVFFASSLYKKWLFGETGCEFYAFCGAVFGITSMITLTAIAM**DRIY**RIFEQGKRSVTPPPFISPTSQVLLGVWLYALAWSLPPFFGWSAYVPEGLLTSCSWDYMTFTPQVRAYTMLLFCFVFFLPLLIIIFCYIFIFRARGVPETFNEAKVALIVILLFVLSWAPYSTVALVAFAGYSHILTPYMSSVPAVIAKASAIHNPIIYAITHPEQNVQKRKRSLKKTSTMAAPPKSENSEDAK
